# Furin-responsive triterpenine-based liposomal complex enhances anticervical cancer therapy through size modulation

**DOI:** 10.1080/10717544.2020.1827086

**Published:** 2020-11-12

**Authors:** Yunyan Chen, Mengfei Guo, Ding Qu, Yuping Liu, Jian Guo, Yan Chen

**Affiliations:** a Affiliated Hospital of Integrated Traditional Chinese and Western Medicine, Nanjing University of Chinese Medicine, Nanjing, China; b Jiangsu Provincial Academy of Traditional Chinese Medicine, Nanjing, China; c School of Pharmacy，Wannan Medical College, Wuhu, China

**Keywords:** Tripterine, microemulsion, liposome, penetration, accumulation, anticervical cancer

## Abstract

The accumulation and penetration of antitumor drugs in tumor tissues are directly related to their antitumor effects. The particle size of the nanodrug delivery system is one of the most important factors for the accumulation and penetration of antitumor drugs within tumor tissues. Generally, nanodelivery systems of intermediate size (100–120 nm) are capable of efficient accumulation owing to prolonged circulation and enhanced permeability and retention (EPR) effect; however, smaller ones (20–40 nm) are effective for deep penetration within tumor tissue. Currently a conventional drug delivery system cannot possess two types of optimal sizes, simultaneously. To solve this and to enhance cervical cancer treatment, a furin-responsive triterpenine-based liposomal complex (PEGcleavable Tf-CTM/L), with Tf-CTM (transferrin-modified tripterine-loaded coix seed oil microemulsion) in core, coated with a thermo-sensitive lipid and a kind of PEG shell modified with a furin-cleavable peptide was developed to improve tumor-specific accumulation and penetration. Herein, PEGcleavable Tf-CTM/L was capable of efficient accumulation because of EPR effect. The PEG shells could timely detach under stimulation of overexpressed furin protein to solve the problem of the steric hindrance dilemma. The small-sized Tf-CTM released under stimulation of tumor microthermal environment in cervical cancer, which was efficient with regards to deep penetration at tumor sites. Notably, compared to the use of triterpenine alone, PEGcleavable Tf-CTM/L promoted anticervical efficacy and displayed diminished systemic toxicity by efficient accumulation and deep penetration of antitumor drugs within tumor tissues. Our study provides a new strategy, and holds promising potential for anticervical cancer treatment.

## 1. Introduction

Cervical cancer, one of the most common malignancies, threatens women’s lives and health worldwide (Banerjee & Kamrava, 2014; Vaisy et al., 2014; Tsikouras et al., 2016; Gaffney et al., 2018). In recent years, although the human papillomavirus (HPV) prophylaxis vaccine has been developed to prevent cervical cancer, it has not been widely used in developing countries. This is mainly due to limitations in regional economic development (Shi et al., 2008; Colombara and Wang, 2013; Zeng et al., 2016; Yin, 2017). In the clinic, a combination of carboplatin and paclitaxel has been widely used, for decades, as the gold standard for cervical cancer treatment to improve the quality of life and prolong patient survival (Mabuchi et al., 2009; Angioli et al., 2014; Symonds et al., 2015; Yang et al., 2015). However, the rapid clearance of antitumor drugs in the physiological circulation, and common side effects leads to the reduced efficacy of cervical cancer treatment. The clinical treatment of cervical cancer still faces great challenges.

Fortunately, nanodrug delivery systems have significantly improved the efficacy of anti-tumor drugs, while reducing the side effects of nontargeted drug delivery systems (Akhter et al., 2013; Biswas et al., 2016; Tran et al., 2017; Zhen et al., 2017; Reinišová et al., 2019; Sun et al., 2019). However, the efficiency of nanodrug delivery systems still significantly affects the efficacy of anti-tumor treatment. Whether the delivery system could efficiently accumulate at tumor sites and deeply penetrate within tumor tissues is an obstacle that needs to be overcome. The particle size of a nano-sized drug delivery system is the most crucial factor for accumulation and penetration in tumor tissues (Ding et al., 2012; Wang et al., 2015; Scenario, 2016; Islam et al., 2017; Zhang et al., 2017; Ruan et al., 2019; Yu et al., 2019; Tang et al., 2013).

**Scheme 1.**
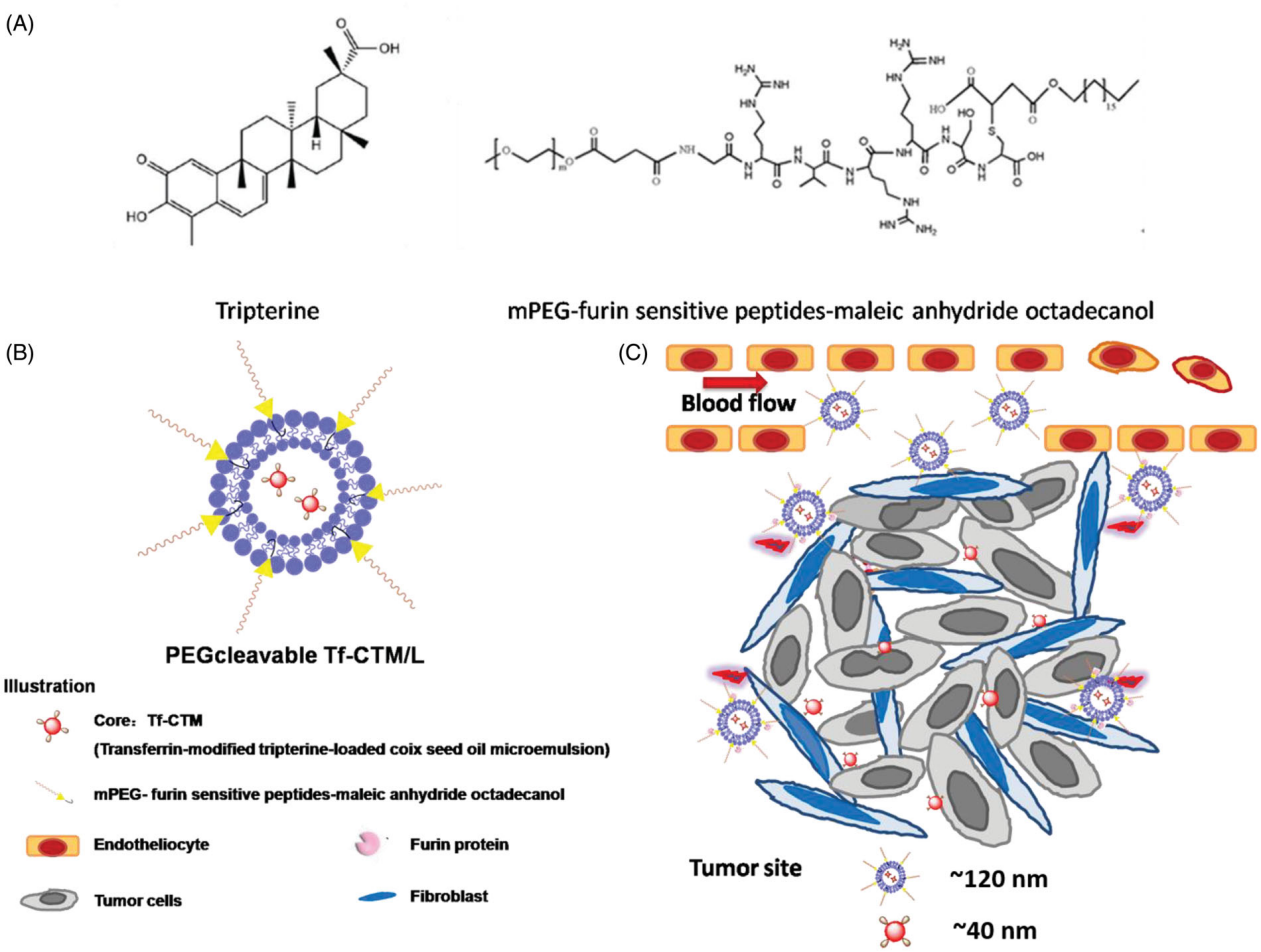
(A) Chemical structure of tripterine and furin-cleavable PEG shell. (B) and (C) Illustration of PEGcleavable Tf-CTM/L (As previous reported, coix seed oil acted as an oil phase, as well as an antitumor ingredient, tripterine as an antitumor component, and Tf as an active targeting ligand was modified on the surface of Tf-CTM. PEGcleavable Tf-CTM/L coated with thermo-sensitive lipid and a kind of PEG shell modified with furin-cleavable peptide was developed for improved tumor-specific accumulation and penetration to enhance cervical cancer treatment). (Chen et al., 2018).

To overcome this obstacle, we developed a furin-responsive triterpenine-based liposomal complex (PEGcleavable Tf-CTM/L) capable of cleavage by using a programmed assembly strategy that triggered by the microenvironment of cervical cancer models such as the overexpression of furin protein (Scheme 1). Tf-CTM was coated with thermo-sensitive lipids and PEG shell modified with a furin-cleavable peptide (S1), and PEGcleavable Tf-CTM/L with intermediate size (∼110 nm) was capable of efficient accumulation. Owing to enhanced permeability and retention (EPR) effect, PEG shells can predominantly improve drug accumulation at tumor sites (Zhao et al., 2016; Hardiansyah et al., 2017; Ibrahim et al., 2017; Mcmasters et al., 2017; Kenechukwu et al., 2018; Nam et al., 2018; Rahmatolahzadeh et al., 2018). However, PEG shells can reduce cellular uptake due to steric hindrance (Grosse et al., 2010; Zhu et al., 2014; Jiang et al., 2017). To overcome this steric hindrance dilemma, the PEG shell was modified with a furin-cleavable peptide and its detachment was characterized using quartz crystal microbalance (QCM) technology (Wen et al., 2012; Wang et al., 2013; Yu et al., 2013; Jin et al., 2015). After efficient accumulation at tumor sites, the PEG shells could automatically detach from the liposomal complex under the stimulation of overexpressed furin protein. Thereafter, the lipid shells could become unstable in the tumoral microthermal environment, to perform deep tumor penetration by small-sized Tf-CTM (∼40 nm) (Kumar et al., 2010; Jaaks & Bernasconi, 2017). For small-sized Tf-CTM, we developed a dual-component microemulsion delivery system (Tf-CTM) comprising tripterine (anticancer drug 1) and coix seed oil (anticancer drug 2, oil phase). As previously reported, it has been verified that after transferrin modification, Tf-CTM exhibited enhanced anti-cervical cancer treatment (Qu et al., 2015, 2017; Chen et al., 2018; Qu et al., 2018).

Thus, PEGcleavable Tf-CTM/L possessed two types of optimal sizes, simultaneously; offering a new insight into effectively improving the antitumor efficacy of the nanodrug delivery system by resolving the contradiction of optimal size of accumulation and penetration at the tumor sites.

## 2. Materials and methods

### 2.1. Chemicals and reagents

GRVRRSC (G: glycine; R: arginine; V: valine; S: serine; C: cysteine) was provided by GL Biochem Co., Ltd. (Shanghai, China). MonomethoxyPEG (mPEG) was provided by Sigma-Aldrich (St. Louis, MO). 4-Dimethylaminopyridine (DMAP), 1, 1-dioctadecyl-3, 3, 3, 3-tetramethylindodicarbo cyanine iodide (DiD) and Triethylamine (TEA) were all provided by Aladdin Co., Ltd. (Shanghai, China). Octadecanol, succinic anhydride, maleic anhydride, and tripterine (purity >98.0%) were all purchased from Sinopharm Chemical Reagent Co., Ltd. (Shanghai, China). Coix seed oil (extracted by carbon dioxide supercritical technology, purity >85%). RH 40 and PEG 400 were purchased from BASF Co., Ltd. (Ludwigshafen, Germany). Dipalmitoylphosphatidylcholine (DPPC), stearoyllysophosphocholine (S-lysoPC), and Distearoylphosphoethanolamine-PEG2000 (DSPE-MPEG_2k_) were provided by A.V.T Co., Ltd. (purity > 98.0%, China). Double-distilled water was purified via Milli Q purification system (Merck Millipore). All other reagents used were of analytical grade.

### 2.2. Animals

Nude mice (BALB/c, 22 ± 2 g) were provided by the Model Animal Research Center of Yangzhou University (Jiangsu, China). All the animals were acclimatized for at least 7 days, and raised under 12 h light/dark cycles with food/water. The protocols were approved by the Animal Experimentation Ethics Committee of Nanjing University of Traditional Chinese Medicine (Nanjing, China).

### 2.3. Preparation and characterization of PEGcleavable Tf-CTM/L

The preparation of PEGcleavable Tf-CTM/L was carried out in two steps as follows. Firstly, the main anti-tumor microemulsion, Tf-CTM comprising tripterine (10 mg), coix seed oil (400 mg), RH 40 (450 mg), PEG 400 (150 mg), and Tf was prepared according to our previous report (Chen et al., 2018). Secondly, by the thin-film hydration method, Tf-CTM was then encapsulated into the hydrophilic core of the liposome.45 18.00 mg of DPPC, 4.00 mg of DSPE-mPEG2k, 1.60 mg of S-lysoPC, and 2.36 mg of furin-cleavable peptide were dissolved in chloroform. The chloroform was removed to acquire the lipid film, at 37 °C under reduced pressure condition. And then the lipid film was hydrated with 10 mL Tf-CTM using a rotary evaporator at 50 °C (Qu et al., 2018). The obtained PEGcleavable Tf-CTM/L was successively ultrasonicated (250 W, ultrasound 3 s interval 2 s for 10 min), extruded through a membrane filter (0.45 μm), and finally purified using G-50 Sephadex column.45 Tf-CTM/L was prepared without furin-cleavable peptide. DSPE-mPEG5k was used, instead of the furin-cleavable peptide, to prepare PEGuncleavable Tf-CTM/L as a control.

The surface potential and average particle size of PEGcleavable Tf-CTM/L were measured by DLS (Nano ZS, Malvern Instruments Ltd, Malvern, UK). The morphology of PEGcleavable Tf-CTM/L was characterized by TEM (JEM-200CXJEOL, Tokyo, Japan) (Qu et al., 2015, 2017; Chen et al., 2018; Qu et al., 2018).

### 2.4. Drug encapsulation efficiency (EE) of PEGcleavable Tf-CTM/L

The EE was calculated as follows:

$${\text{EE}}_{\text{tripterine}}\left(\%\right)\;=\;W_{\text{encapsulated}\;\text{drug}}/W_{\text{feeding}\;\text{drug}}\times \;\%$$

where *W* represents the tripterine amount of various formulations.

The content of tripterine in PEGcleavable Tf-CTM/L was determined by HPLC (1260 Infinity, Agilent Technologies) at 426 nm. The chromatographic conditions were as follows: C18 column (4.6 × 150 mm × 5 µm, Diamond); mobile phase (methanol: water = 90:10); column temperature (30 °C); flow rate (1.0 mL/min) (Qu et al., 2015, 2017; Chen et al., 2018; Qu et al., 2018).

### 2.5. Drug release *in vitro*

As previously reported, tripterine released from PEGcleavable Tf-CTM/L was determined via dialysis method (Qu et al., 2015, 2017; Chen et al., 2018; Qu et al., 2018). 1 mL of PEGcleavable Tf-CTM/L was firstly transferred to a dialysis bag (10 kDa MWCO), and then followed by incubation within 120 mL of PBS (pH 7.4) with 0.5% (wt%) Tween 80, under pH 7.4 at 37 °C and 42 °C. During the 0–24 h period, 50 μL of PEGcleavable Tf-CTM/L solution was taken at the predetermined time intervals with rotary shaking at 60 rpm. The concentration and the accumulative release of tripterine from PEGcleavable Tf-CTM/L was determined via HPLC.

### 2.6. Mechanism of PEGdetachment studied by quartz crystal microbalance (QCM)

The characterization of the furin-cleavable PEG shell of PEGcleavable Tf-CTM/L was studied by QCM (Pang et al., 2010; Kang et al., 2014; Qian et al., 2016; Marsh et al., 2018). In this part of the study, the QCM experiment was conducted at 25 °C. The frequency change (*ΔF*) was recorded using a third overtone. PEGcleavable Tf-CTM/L and PEGuncleavable Tf-CTM/L were employed to investigate the furin-cleavable PEG shell. First, PEGcleavable Tf-CTM/L and PEGuncleavable Tf-CTM/L were dissolved within PBS (pH 7.4) and injected into QCM-D cells until the frequency was constant and dissipation for adsorption to the films. PBS (pH 7.4) and furin protein solution were injected successively for 1 h, and the change in frequency was observed.

### 2.7. Cell culture and preparation of 3D tumor spheroids

HeLa cells were cultured in Dulbecco’s modified Eagle’s medium (DMEM, 10% FBS, 100 μg/mL streptomycin/penicillin) under 5% CO_2_. HeLa cells (1 × 10^3^) were seeded in the 96-well plates with the surface of an agarose-based culture medium, under an atmosphere of 5% CO_2_ at 37 °C. HeLa 3 D tumor spheroids with an appropriate size (∼300 nm) were selected and then transferred to confocal dishes, for study after 10 days of incubation (Qu et al., 2015, 2017; Chen et al., 2018; Qu et al., 2018). HeLa 3D tumor spheroids were treated with CTM, Tf-CTM with small size and Tf-CTM/L, PEGcleavable Tf-CTM/L with large size at fluorescein isothiocyanate (FITC, 10 µM) for 8 h and then fixed with 4% (v%) paraformaldehyde and then observed via CLSM (Z-stack tool, 5 µm interval/scan).

### 2.8. Intracellular delivery and cellular uptake

HeLa cells (1 × 10^5^) were seeded in six-well plates and treated with FITC-labeled PEGcleavable Tf-CTM/L for 2 h after reaching 80% of the overspread. The concentration of various formulations was calculated as FITC (10 µM). After incubation, HeLa cells were rinsed with PBS three times, and then collected by trypsin without EDTA to harvest in 0.2 mL of PBS via flow cytometry (Guava 6HT, Merck Millipore).

According to previous studies, the intracellular delivery of various tripterine treatments was studied (Qu et al., 2015, 2017; Chen et al., 2018; Qu et al., 2018). HeLa cells (1 × 10^5^) were seeded in 12-well plates and treated with FITC-labeled PEGcleavable Tf-CTM/L at a FITC concentration of 10 µM. After 2 h of incubation, HeLa cells were washed by PBS (thrice, ice cold), followed by staining with MitoTracker Red (100 nM, Yeasen, China) and LysoTracker Red (50 nM, Abcam, UK). Finally, HeLa cells were fixed via 4% (v%) paraformaldehyde (25 °C) and observed by CLSM.

### 2.9. Cytotoxicity

Five thousand HeLa cells were seeded in 96-well plates and cultured for 24 h. HeLa cells underwent various treatments for 24 h at predetermined concentrations (0.625 − 20 μg/mL) after removal of DMEM. After treatment, 5 μL of MTT solution was then added and HeLa cells were stained for further 4 h. The formazan crystals obtained were dissolved in DMSO (100 µL). The absorbance (A) was measured using a microplate reader at 570 nm (Qu et al., 2015; 2017; Chen et al., 2018; Qu et al., 2018).

### 2.10. Cell apoptosis induction

According to a previous report, HeLa cell suspension (50 µL, containing 5 × 10^3^ cells) was collected and then incubated with Annexin V-PE staining kit (50 µL, Guava, Merck Millipore) for 15 min (Qu et al., 2015, 2017; Chen et al., 2018; Qu et al., 2018). The tripterine concentrations of various formulations were 1 μg/mL, the incubation time was 2–6 h, and the analysis was performed, immediately, by flow cytometry.

### 2.11. Xenograft tumor models

HeLa cell suspension (200 μL, containing 2 × 10^7^ cells) was injected to the right hind leg of the nude mice subcutaneously, to establish the HeLa xenograft tumor model. The tumor size was measured via vernier calipers and calculated using the following formula: *V* = (*L* × *W*^2^)/2, where *W* represents a smaller vertical width and L represents a larger vertical length (Qu et al., 2018).

### 2.12. In vivo *imaging*

When the tumor volume increased to 120 mm^3^, HeLa xenograft nude mice were randomly divided into four groups and designed as follows: DiD, Tf-DiD-C-MEs, Tf-DiD-C-MEs/L, and PEGlyated Tf-DiD-CMEs/L. 0.2 mL of various formulations was administered intraperitoneally to nude mice at a DiD dose of 30 μg/mL (Qu et al., 2018).

After isoflurane-anesthesia was performed, the near-infrared images of administered nude mice were acquired using an *in vivo* imaging system (PerkinElmer, USA) at predetermined time points, post-administration (Qu et al., 2018). The fluorescence measurement was performed by the region-of-interest (ROI) function, adopting the live image software. Afterward, at 12 h post-treatment, the mice were euthanized. Using an *in vivo* imaging system, fluorescence images of major normal organs, such as the heart, liver, spleen, lung, kidney, and tumor tissues were collected.

### 2.13. Antitumor efficacy and systemic safety

With an average tumor size (−120 mm^3^), HeLa xenograft tumor-bearing nude mice were intraperitoneally injected with saline (negative control), tripterine, Tf-CTM, Tf-CTM/L-37 °C, Tf-CTM/L-42 °C, PEGcleavable Tf-CTM/L-37 °C, PEGcleavable Tf-CTM/L-42 °C, and PEGuncleavable Tf-CTM/L-42 °C at a tripterine dose of 1.5 mg/kg once every 2 days. The efficacy of Tf-CTM/L-42 °C, PEGcleavable Tf-CTM/L-42 °C and PEGuncleavable Tf-CTM/L-42 °C were also assessed 6 h after administration by immersing the tumor-bearing leg in a 42 °C water bath for 1 h (Al-Jamal et al., 2012). The body temperature of all the other animals was maintained about 37 °C throughout our studies. The body weight and tumor size were daily recorded. Blood samples were assembled from the eyeballs of mice, followed by harvesting major organs, tumor, heart, liver, spleen, lung, and kidney. All blood samples were used for blood route analysis, liver/kidney function, and cytokines/chemokines. The cytokines/chemokines, including interleukin-2 (IL-2), interleukin-6 (IL-6), interleukin-10 (IL-10), interleukin-12A (IL-12A), chemokine 2 (CCL-2), tumor necrosis factor-α (TNF-α), transforming growth factor β1 (TGF-β1), and interferon-γ (IFN-γ). The tumor index (TI) was calculated using the following formula: weight tumor/weight body and the inhibition of tumor growth was calculated using the following formula: 1－(TI_treat_/TI_saline_). Hematoxylin and eosin (HE) staining of harvested organs was performed using standard protocols. Conventional immunohistochemistry was used to observe the cell proliferation of the tumors using the Ki-67 antibody staining method. Furthermore, terminal deoxynucleotidyl transferase dUTP nick end labeling (TUNEL, KeyGen Biotech, China) was used to evaluate tumor apoptosis.

### 2.14. CD-31 and α-SMA assay

The tumor microenvironment was characterized by α-SMA and anti-CD-31 antibody. Prior to α-SMA staining, tumor sections were firstly deparaffinized via dimethylbenzene three times, and then rinsed with water-alcohol solutions, followed by incubation with 0.1% Triton X-100 within PBS (15 min) and finally blocking with 1% BSA (30 min). Thereafter, the tumor sections were incubated with primary polyclonal human α-SMA antibody (Abcam, UK) and then further incubated with Alexa Fluor 555-conjugated secondary antibody both for 1 h. Followed by washing samples with PBS thrice, staining with DAPI for 30 min, and then fixing with 4% paraformaldehyde for 15 min. All of the above operations were carried out at room temperature (25 °C) and observed by CLSM. Likewise, CD-31 staining was similar to that of α-SMA. The primary antibody was changed to CD-31 antibody (Qu et al., 2017; Qu et al., 2018).

### 2.15. Statistical analysis

The results are described as the mean ± standard deviation (SD). Statistical significance was determined using a two-tailed Student's *t*-test. The differences were considered statistically significant at *p < .05, **p < .01.

## 3. Results and discussion

### 3.1. Characterization of PEGcleavable Tf-CTM/L

**Figure 1.**
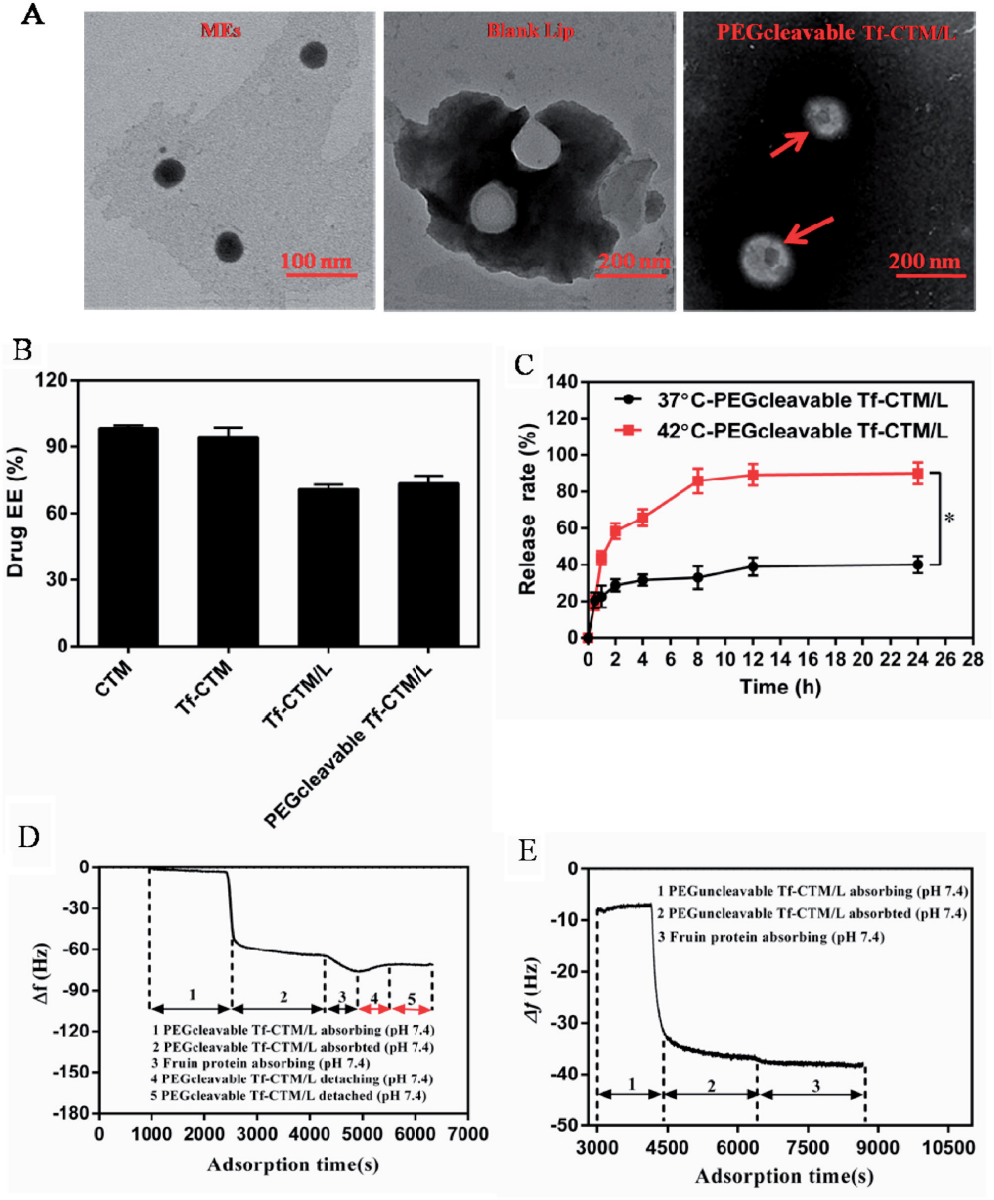
Characterization of PEGcleavable Tf-CTM/L. (A) TEM images of Tf-CTM, blank Lip and PEGcleavable Tf-CTM/L. The red arrows represent the liposome-internal Tf-CTM. (B) Drug encapsulation efficiency of various treatments. (C) *In vitro* accumulative drug release of PEGcleavable Tf-CTM/L under pH 7.4 within 24 h. Data are represented as mean ± SD; *n* = 3. *p < .05. (D) The dynamic process of PEGcleavable Tf-CTM/L adsorption and PEGcleavable Tf-CTM/L detachment monitored by QCM under pH 7.4. (E) The dynamic process of PEGuncleavable Tf-CTM/L adsorption and PEGuncleavable Tf-CTM/L detachment monitored by QCM under pH 7.4.

PEGcleavable Tf-CTM/L was mainly comprised of active targeting Tf-CTM, lipid bilayer membrane, and PEG shell modified with furin-cleavable peptide. The particle sizes of Tf-CTM, Tf-CTM/L, and PEGcleavable Tf-CTM/L were 40.33 ± 0.26, 111.3 ± 1.07, and 115.3 ± 1.37 nm, respectively, (Table 1). As shown in Figure 1(A), Tf-CTM displayed a spherical morphology with a small partical size of approximately 40 nm. Blank Lips (without Tf-CTM as the core) with a clear lipid bilayer were 110 nm around. In particular, PEGcleavable Tf-CTM/L exhibited the structure of Tf-CTM with small particle size located inside the large-sized liposome, illustrating that Tf-CTM was capable of being encapsulated into liposome, using the thin film dispersion method, which results in the liposomal complex simultaneously possessing two optimal sizes (Qu et al., 2018). The zeta potentials of Tf-CTM, Tf-CTM/L, and PEGcleavable Tf-CTM/L were −13.6 ± 1.13, −22.5 ± 0.361, and −47.8 ± 0.591 mV, respectively, (Table 1). In comparison with Tf-CTM, the zeta potential of Tf-CTM/L was significantly increased, demonstrating that Tf-CTM was mainly distributed in the liposome. In comparison with Tf-CTM/L at the corresponding mass ratio, the zeta potential of the PEGcleavable Tf-CTM/L was remarkably higher, which indicated that Tf-CTM/L was mainly successfully modified by the furin-cleavable peptide with negative charge (Qu et al., 2015, 2017; Chen et al., 2018; Qu et al., 2018). The encapsulation efficiency of tripterine in CTM, Tf-CTM, Tf-CTM/L, and PEGcleavable Tf-CTM/L were 98.45, 94.50, 70.81, and 73.40%, respectively. The EE of Tf-CTM/L and PEGcleavable Tf-CTM/L showed a gradually declining trend, which might be due to the increased amounts of excipients in various treatments (Figure 1(B)). According to previous reports, the majority of solid tumors, including cervical cancer, are characterized by micro-thermal properties. The average temperature of the tumor site was nearly 42 °C, and the temperature condition was generally chosen to be 42 °C, according to the clinical drug applications (Maruyama et al., 1993; Lindner et al., 2004; Mills and Needham, 2005; Hossann et al., 2010; Li et al., 2010). With the increase in time of 24 h, the accumulative release of tripterine from PEGcleavable Tf-CTM/L were 40.03 and 90.02% under the condition of 37 °C and 42 °C, respectively, suggesting that significant differences existed between the release profile of different temperature (Figure 1(C)). More efficient release of tripterine under tumoral microthermal environment (42 °C) could enhance antitumor efficacy, while lower leakage of tripterine under body temperature (37 °C) could reduce systemic toxicity. QCM results indicated that the PEG modified with furin-cleavable peptide could effectively detach from PEGcleavable Tf-CTM/L under the stimulation of overexpressed furin protein (Figure 1(D), phase 4), while there was no detachment observed from PEGuncleavable Tf-CTM/L (Figure 1(E)). In addition, the detachment of the PEG shell could further solve the PEG-steric hindrance dilemma to promote cellular uptake of tripterine and enhance the efficacy of cervical cancer treatment.

**Table 1.** Particle size and zeta potential of various tripterine treatments (*n* = 3).

Formulation	Size (nm)	PDI	Zeta potential (mV)
Tf-CTM	40.33 ± 0.26	0.146 ± 0.01	−13.63 ± 1.13
Tf-CTM/L	111.3 ± 1.07	0.311 ± 0.05	−22.5 ± 0.361
PEGcleavable Tf-CTM/L	115.3 ± 1.37	0.281 ± 0.15	−47.8 ± 0.591
PEGcleavable Tf-CTM/L (incubted with furin protein)	100.6 ± 2.01	0.491 ± 0.26	−30.2 ± 0.328

### 3.2. Cellular uptake assay

**Figure 2.**
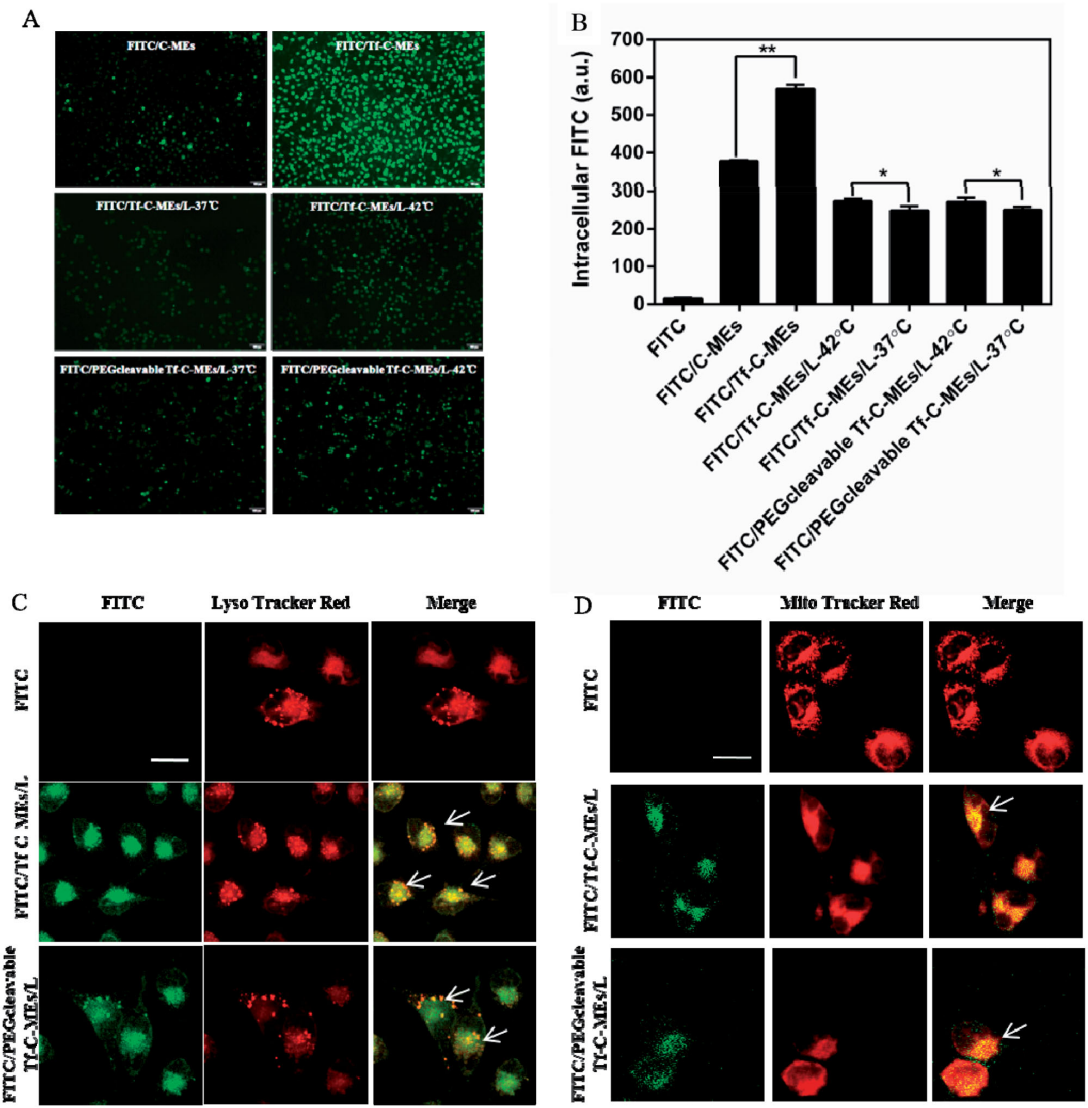
Cellular uptake and intracellular delivery (A) Fluorescent images of HeLa cells incubated with FITC- labeled various formulations for 2 h. Scale bar is 100 μm. (B) Mean fluorescene intensity of HeLa cells were analyzed by Flow cytometry after incubation with preparations for 2 h. Data are represented as mean ± SD, *p < .05, **p < .01. (C) Intracellular delivery of FITC/Tf-C-MEs/L and FITC/PEGcleavable Tf-C-MEs/L within HeLa cells observed using CLSM. The arrows represent formulation entrapped in the endo/lysosomes. Scale bar is 25 μm. (D) Intracellular delivery of FITC/Tf-C-MEs/L and FITC/PEGcleavable Tf-C-MEs/L within HeLa cells observed using CLSM. The arrows represent formulation entrapped in the mitochondria. Scale bar is 25 μm.

We evaluated the uptake of various formulations in cellular uptake study. The intracellular fluorescence of the FITC/Tf-C-MEs was more intense than that of various treatments group (Figure 2(A,B)), suggesting the enhancement of endocytosis after transferrin modification (**p < .01) (Chen et al., 2018). The intracellular fluorescence of the 42 °C group was more intensive compared with that of the 37 °C group (*p < .05), suggesting that enhancement of endocytosis was triggered by temperature (Chen et al., 2018). In particular, the uptake fluorescence intensity of HeLa cells by the FITC/Tf-C-MEs was 568.97 ± 11.42, and that of FITC/PEGcleavable Tf-C-MEs/L-42 °C was 271.68 ± 11.58, indicating an inherent advantage of small-sized nanoparticles on internalization.

Intracellular localization of various formulations was detected by laser confocal microscopy (Figure 2(C)). Various formulations were labeled with FITC (green fluorescence) and LysoTracker Red was used as the fluorescent probe for endo/lysosomes (red fluorescence). Based on our previous report, cells treated with FITC/Tf-C-MEs showed significant yellow fluorescence, suggesting that FITC/Tf-C-MEs were probably retained by endo/lysosomes (Qu et al., 2015, 2017; Chen et al., 2018; Qu et al., 2018). Both FITC/Tf-C-MEs/L and FITC/PEGcleavableTf-C-MEs/L showed co-localization in lysosomes (yellow fluorescence), which may be due to the release of FITC/Tf-C-MEs.

Meanwhile, tripterine can inhibit the proliferation of tumor cells through the mitochondrial targeting pathway (Yoon et al., 2014; Shweta et al., 2015; Yu et al., 2015). Mitochondria were stained with MitoTracker Red (red fluorescence) and formulations were labeled with FITC (green fluorescence) (Chen et al., 2018). Interestingly, we found colocalization in the mitochondria of FITC/Tf-C-MEs, FITC/Tf-C-MEs/L, and FITC/PEGcleavable Tf-C-MEs/L, suggesting that FITC/Tf-C-MEs were probably retained by mitochondria as well (Figure 2(D)).

### 3.3. Cell apoptosis induction

For the HeLa cell apoptosis study, the concentration of tripterine was 1 μg/mL and the incubation time was 2, 4, and 6 h, respectively.

**Figure 3.**
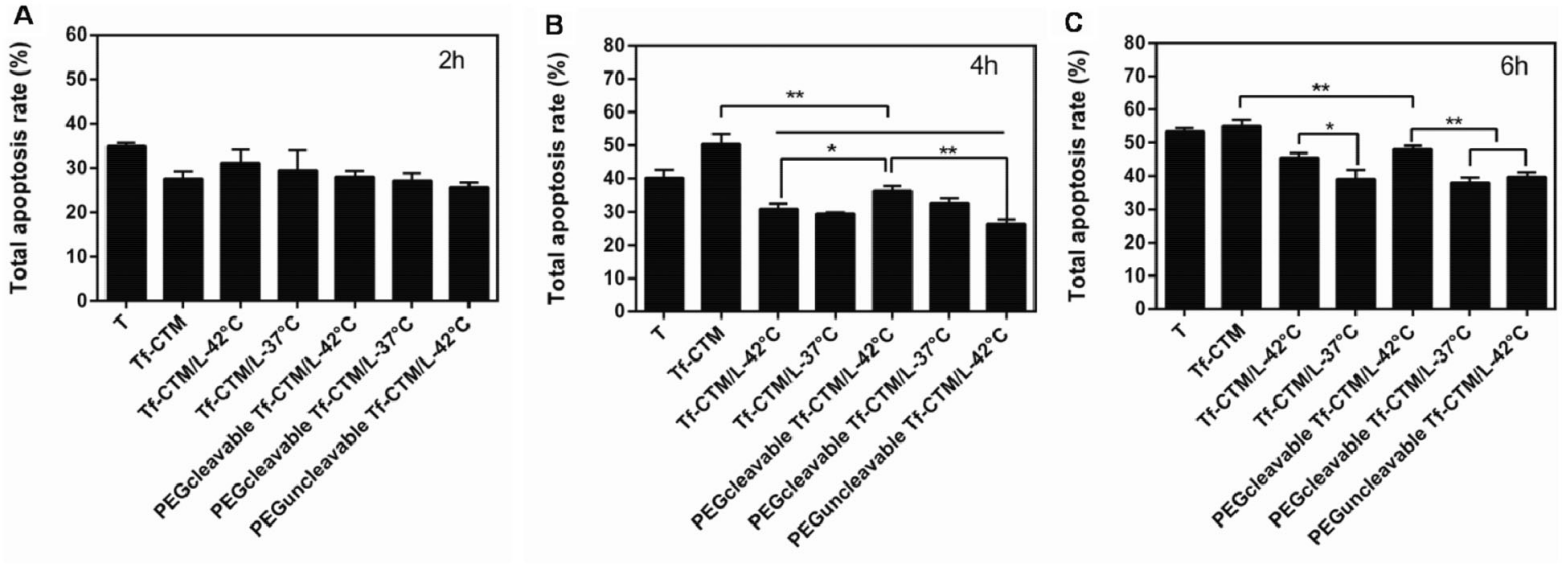
Quantitative analysis of total apoptosis rate of HeLa cells induced by various treatments (Data are represented as mean ± SD, **p < .01, *p < .05, *n* = 3). Concentration of tripterine was 1 μg/mL and the administration time was for 2 h (A), 4 h (B), 6 h (C).

After treatment for 2 h, tripterine exhibited the strongest ability to induce apoptosis, and the apoptosis rate was 34.92% (Figure 3(A)). The apoptosis rate of various treatments was lower than that of tripterine, mainly due to the dual encapsulation of liposomes and microemulsions. The dual-encapsulation structure can delay tripterine release from PEGcleavable Tf-CTM/L and thus reduce the toxicity against nontargeted sites.

After treatment for 4–6 h, there was a significant difference between PEGcleavable Tf-CTM/L-42 °C (36.30%, 48.07%) and PEGcleavable Tf-CTM/L-37 °C (32.72%, 37.94%) in the total apoptosis rate, which suggests that enhancement of cellular uptake was triggered by temperature. Compared to PEGcleavable Tf-CTM/L-42 °C (36.30%), Tf-CTM (50.35%) exhibited 1.38-fold higher apoptosis rates at 4 h, and 1.14-fold higher apoptosis rates at 6 h, respectively, (Figure 3(B,C)), which suggested that small size and modification of transferrin could enhance apoptosis of tumor cells (Chen et al., 2018).

Interestingly, we found that Tf-CTM significantly induced cell apoptosis. Meanwhile, the apoptosis rate of PEGcleavable Tf-CTM/L-42 °C showed a gradually increasing trend as the delivery time increased, suggesting that PEGcleavable Tf-CTM/L-42 °C had a strong potential to induce HeLa cell apoptosis. After treatment with various treatments (1.0 μg/mL of tripterine) for 2, 4, and 6 h. PEGcleavable Tf-CTM/L-42 °C displayed an advantage in apoptosis induction, as shown in Supplementary Figure S2A, B, and C.

### 3.4. Antiproliferative effects *in vitro*

**Figure 4.**
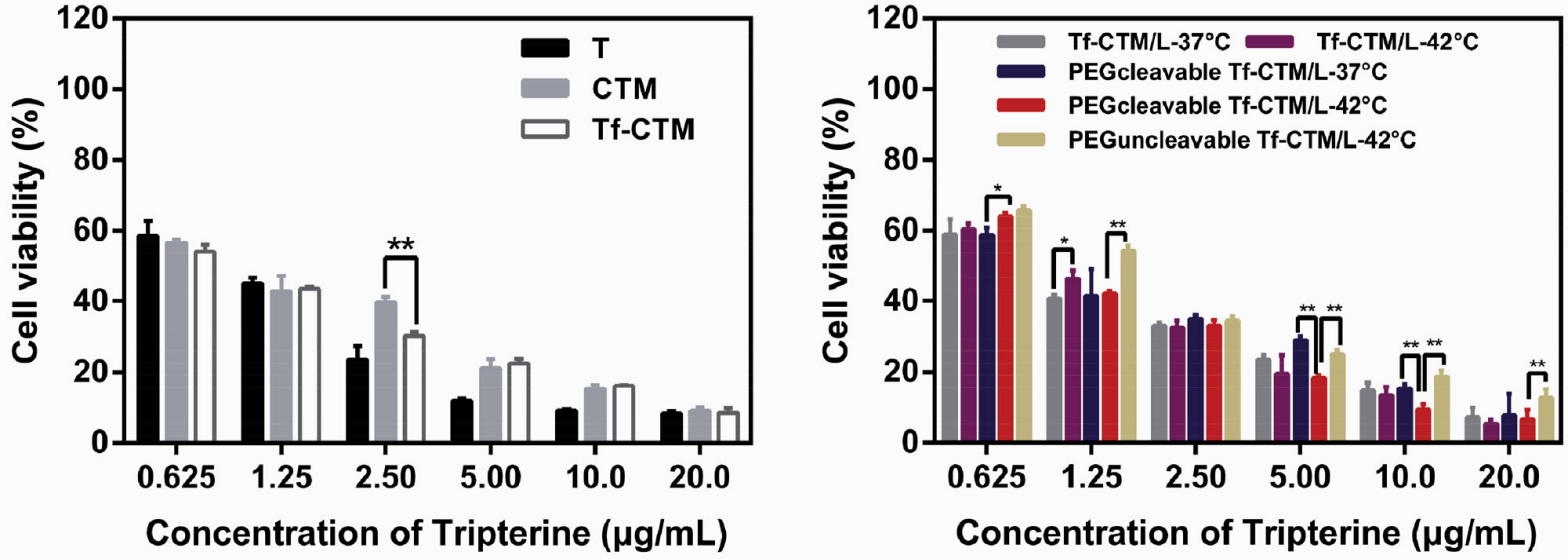
Antiproliferative effects of various treatments against HeLa cells for 24 h by MTT method (Data are represented as mean ± SD, *n* = 6, **p < .01, *p < .05).

After 24 h of treatment with various tripterine formulations, as the concentration of tripterine was higher over than 2.50 μg/mL, the proliferation of HeLa cells was significantly inhibited (Figure 4). The IC_50_ values of various tripterine formulations against HeLa cells were 1.3710 ± 0.02, 0.9524 ± 0.01, 0.8119 ± 0.02, 1.0440 ± 0.01, 0.9192 ± 0.01, 1.0520 ± 0.04, 0.9601 ± 0.02, and 1.378 ± 0.12 μg/mL, respectively. Different tripterine microemulsions such as Tf-CTM can increase the solubility of tripterine and thus improve the uptake of tripterine by HeLa cells. Compared with PEGcleavable Tf-CTM/L-37 °C, PEGcleavable Tf-CTM/L-42 °C exhibited stronger cytotoxicity against HeLa cells. Tf-CTM released by liposome after incubation (42 °C) also presented a more potent cytotoxicity against HeLa cells.

### 3.5. Tumor penetration and treatment

As previously reported, we successfully cultivated HeLa 3D tumor spheroids (Chen et al., 2018). In this study, HeLa three-dimensional (3D) tumor spheroids were also adopted to investigate the intratumor penetration of PEGcleavable Tf-CTM/L.

**Figure 5.**
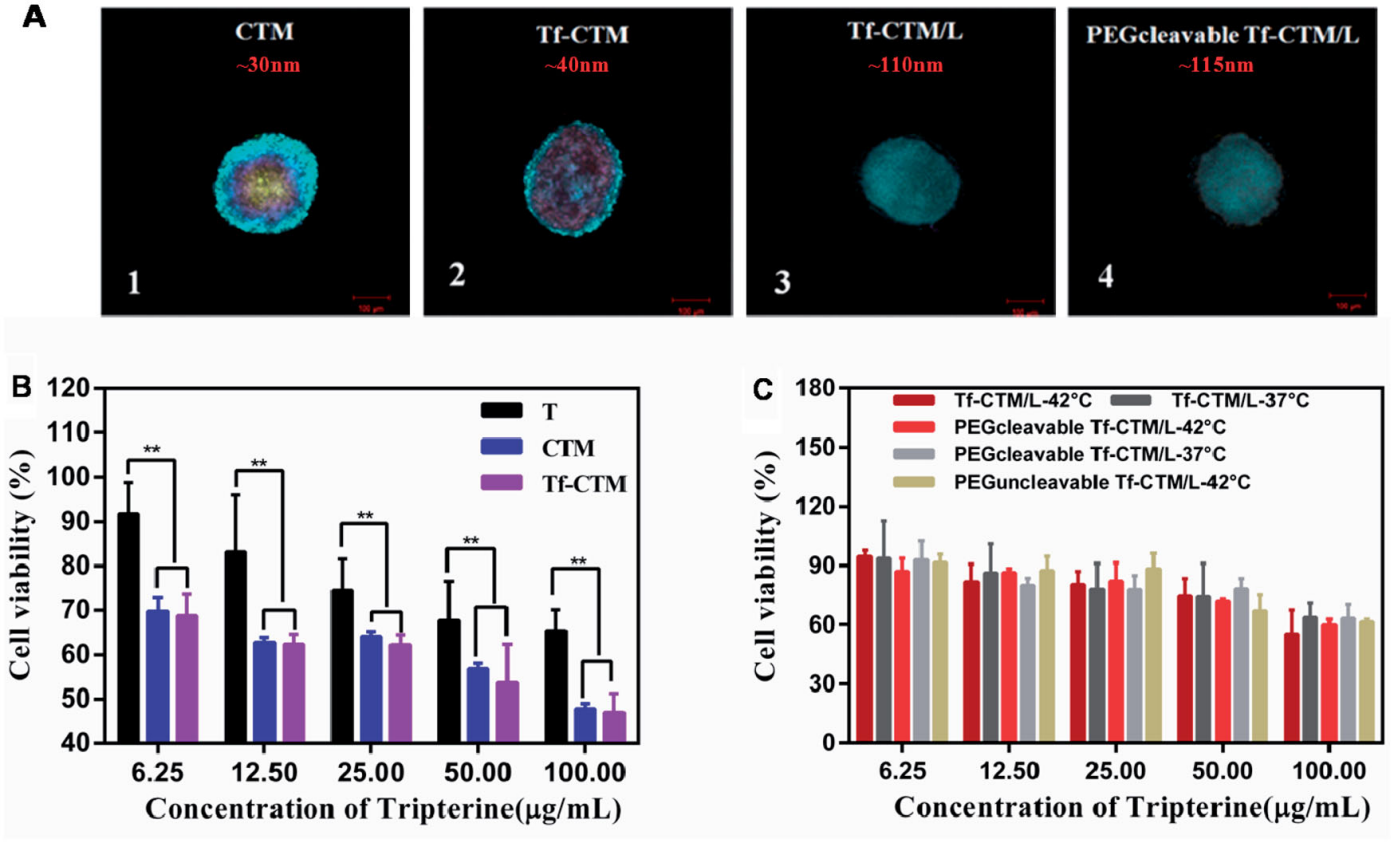
(A) *In vitro* penetration of FITC into the HeLa 3D tumor spheroids after incubation with CTM, Tf-CTM, Tf-CTM/L, PEGcleavable Tf-CTM/L for 8 h. Z-stack images were obtained by CLSM. Scale bar is 100 μm. (1) CTM, (2) Tf-CTM, (3) Tf-CTM/L, (4) PEGcleavable Tf-CTM/L (B) Viability of HeLa 3D tumor spheres treated with T, CTM, Tf-CTM after 24 h using CCK8 (*n* = 6). **p < .01. (C) Viability of HeLa 3D tumor spheres treated with Tf-CTM/L and PEGcleavable Tf-CTM/L after 24 h using CCK8 (*n* = 6).

HeLa 3D tumor spheroids were all compact and spherical after culture for 10 days, with a diameter of approximately 300 μm. The penetration of CTM, Tf-CTM, Tf-CTM/L, and PEGcleavable Tf-CTM/L in HeLa 3D tumor spheroids was investigated after treatment for 8 h (Figure 5(A)). Drug penetration was marked with blue fluorescence. The penetration of various treatments in HeLa 3D tumor spheres was obviously related to the particle size. Small-sized formulations such as CTM (32.47 ± 0.26 nm) and Tf-CTM (40.33 ± 0.26 nm) could penetrate into the interior of HeLa 3D tumor spheres, however, Tf-CTM/L (111.30 ± 1.07 nm) and PEGcleavable Tf-CTM/L (115.30 ± 1.37 nm) with large size just reached the surface around the HeLa 3 D tumor spheres. The permeability of CTM and Tf-CTM with small size was significantly deeper than that of Tf-CTM/L and PEGcleavable Tf-CTM/L with large size (Wilhelm et al., 2016). We further investigated the cytotoxicity of various treatments against HeLa 3 D tumor spheroids (Figure 5(B,C)) (Chen et al., 2018). The IC_50_ values of T, CTM, Tf-CTM, Tf-CTM/L-37 °C, Tf-CTM/L-42 °C, PEGcleavable Tf-CTM/L-37 °C, PEGcleavable Tf-CTM/L-42 °C, and PEGuncleavable Tf-CTM/L-42 °C were 260.2 ± 1.3, 100.7 ± 1.1, 79.60 ± 1.1, 327.6 ± 1.5, 202.0 ± 1.2, 236.9 ± 1.5, 150.6 ± 1.1, and 165.6 ± 2.8 μg/mL, respectively, against HeLa 3D tumor spheroids. We observed dramatically lower cytotoxicity among CTM, Tf-CTM, and PEGcleavable Tf-CTM/L-42 °C, respectively, against HeLa 3D tumor spheroids. The advantage of microemulsion was more obvious than that of the composite, which is related to the deep penetration advantage of nanoparticles with small particle size and the higher uptake of tripterine by HeLa 3D tumor spheroids.

### 3.6. Biodistribution

**Figure 6.**
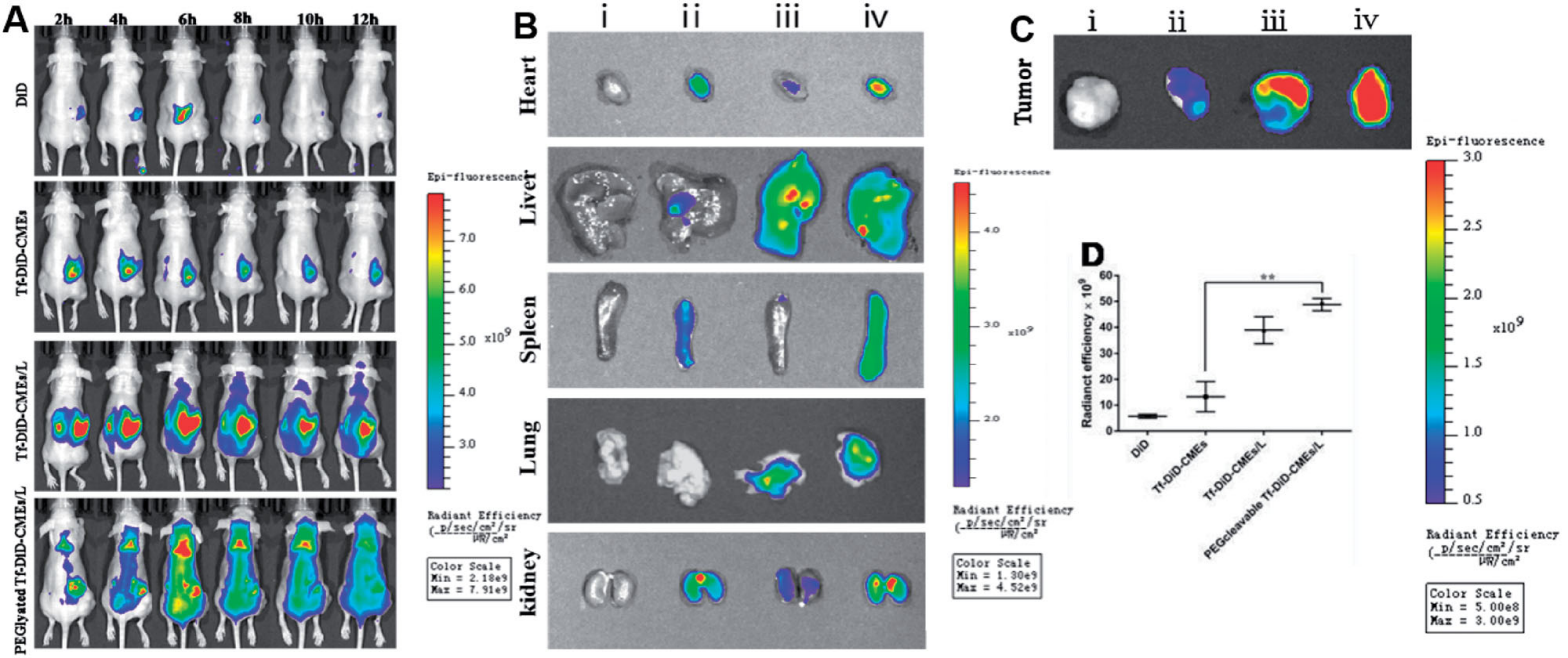
Investigation of biodistribution (A) Distribution of NIR signal on mice treated with different DiD-labeled formulations at the predetermined intervals. (B) Fluorescence images of normal organs at 12 h post-injection. (C) Fluorescence images of tumor tissues at 12 h post-injection. (D) Quantitative analysis of fluorescence in the tumor tissues after 12 h of the administration. Data are represented as mean ± SD, *n* = 3. **p < .01. (i), (ii), (iii), (iv) represent DiD, Tf-DiD-CMEs, Tf-DiD-CMEs/L, and PEGlyated Tf-DiD-CMEs/L, respectively.

To elucidate the biodistribution under NIR *in vivo* imaging system, PEGcleavable Tf-CTM/L was labeled with DiD (PEGlyated Tf-DiD-CMEs/L), and DiD, Tf-DiD-CMEs, and Tf-DiD-CMEs/L acted as controls (Qu et al., 2018). DiD group showed no obvious aggregation at the tumor site during the entire post-injection period. After treatment with Tf-DiD-CMEs, it accumulated at tumor sites. Additionally, the extension of administration time during 1–12 h resulted in the accumulation of Tf-DiD-CMEs at tumor sites becoming more obvious. After treatment with Tf-DiD-CMEs/L around 2 − 12-h post-treatment, strong fluorescence signals were detected around the tumor site. Notably, after treatment with PEGlyated Tf-DiD-CMEs/L, there was a significant NIR signal at the tumor site during the entire observation period (Figure 6(A)). After 12-h post-treatment, PEGlyated Tf-DiD-CMEs/L, and Tf-DiD-CMEs/L mainly accumulated in the liver and detected in the kidney, suggesting the capture of nanoparticles, mainly by the reticuloendothelial system and kidney-mediated elimination (Figure 6(B)). The tumor tissues were harvested to analyze the fluorescence intensity qualitatively and quantitatively to evaluate the potential tumor-targeting ability (Figure 6(C,D)). There was a significant difference between PEGlyated Tf-DiD-CMEs/L and Tf-DiD-CMEs in biodistribution (**p < .01). Notably, tumors from mice treated with PEGlyated Tf-DiD-CMEs/L displayed the highest intensity of fluorescence compared to the others, suggesting that PEGlyated Tf-DiD-CMEs/L was capable of efficient accumulation at tumor sites by the advantage of the EPR effect.

### 3.7. Evaluation of antitumor efficacy

The antitumor efficacy *in vivo* of PEGcleavable Tf-CTM/L-42 °C was investigated, with saline, tripterine, Tf-CTM, Tf-CTM/L-37 °C, Tf-CTM/L-42 °C, PEGcleavable Tf-CTM/L-37 °C, and PEGuncleavable Tf-CTM/L-42 °C as controls. The mice were intraperitoneally administrated with various treatments at doses of 1.5 mg tripterine/kg once every 2 days. Compared to the saline group, the tumor growth of mice treated with various tripterine treatments were largely inhibited (Figure 7(A)). Due to the long circulation of PEG and temperature triggers release of tripterine, PEGcleavable Tf-CTM/L with water bath of 42 °C presented the strongest antitumor activity than any other formulations. Notably, the tumor inhibition ratio of PEGcleavable Tf-CTM/L-42 °C was 80.33%, which was 1.25- and 1.29-fold higher than Tf-CTM/L-42 °C and PEGcleavable Tf-CTM/L-37 °C, respectively (Figure 7(B)). After treatment with PEGcleavable Tf-CTM/L-42 °C using HeLa xenograft tumor-bearing mice, the tumor weight of PEGcleavable Tf-CTM/L-42 °C was the lowest among the various treatment groups (Figure 7(C)). As previously reported, tripterine can lead to several severe side effects, including a short survival period and a sharp reduction in bodyweight. However, the bodyweight of tripterine and Tf-CTM-treated mice was lower than that of the PEGcleavable Tf-CTM/L group, and only minor fluctuations in body weight of PEGcleavable Tf-CTM/L-42 °C were observed; mainly due to dual-encapsulation by liposomes and microemulsions of tripterine (Figure 7(D)). In HE-stained tumor section among various tripterine treatments, PEGcleavable Tf-CTM/L-42 °C induced the largest necrosis area with tumor cell nucleus disappeared, illustrating an overwhelming antitumor capacity (Figure 7(E)). Compared with the saline group, the Ki-67-positive cells in the Tf-CTM, PEGcleavable Tf-CTM/L-37 °C, and Tf-CTM/L-42 °C treatments were sharply reduced. Notably, only a small amount of brown cells were found in the tumor section of PEGcleavable Tf-CTM/L-42 °C (Figure 7(F)). In various tripterine treatments treated tumor sections, a large number of TUNEL-positive cells were detected. Notably, the green fluorescence in the treatment of PEGcleavable Tf-CTM/L-42 °C was stronger than that in the other treatments (Figure 7(G)). Validated by HeLa xenograft tumor-bearing mice models, all the results above verified that PEGcleavable Tf-CTM/L-42 °C had good antitumor efficacy by resolving a contradiction of conformity in optimal size of accumulation and penetration at the tumor sites.

Figure 7.Antitumor efficacy *in vivo* (A) Changes in tumor volume of mice treated with different treatments. **p < .01, *p < .05. (B) Inhibition rate of tumor growth of mice treated with different formulations at day 24 post-xenograft implantation. **p < .01, ^##^*p* < .01 vs PEGcleavable Tf-CTM/L-42 °C; ^#^*p* < .05 versus PEGcleavable Tf-CTM/L-42 °C. (C) Tumor weight of mice treated with various formulations at the end of the observed period. Data are represented as mean ± SD, *n* = 5. **p < .01, ^#^*p* < .05. (D) Alternations in body weight of mice during the treatment. (E) HE-stained images of the tumor slides of mice after treatments. Scale bar is 100 μm. (F) Immunohistochemical images of tumor sections stained with Ki-67 assay kit after various treatments. The brown represents Ki-67-positive cells. Scale bar is 100 μm. (G) Fluorescence images of tumor section stained with TUNEL assay kit after various treatments. The green represents the DNA fragments of apoptotic cells and the blue represents nucleus stained with DAPI. Scale bar is 50 μm.

**Figure:**
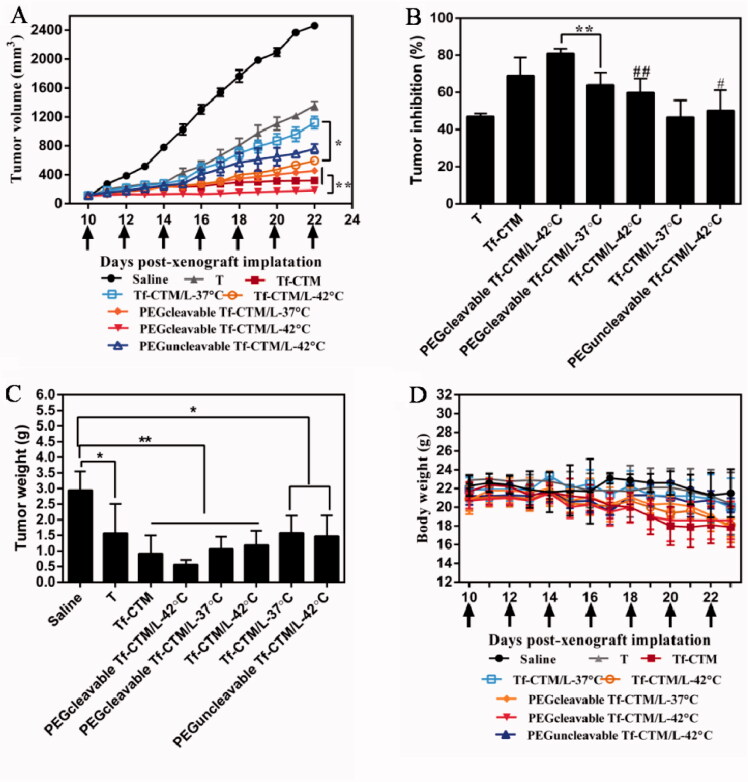

**Figure:**
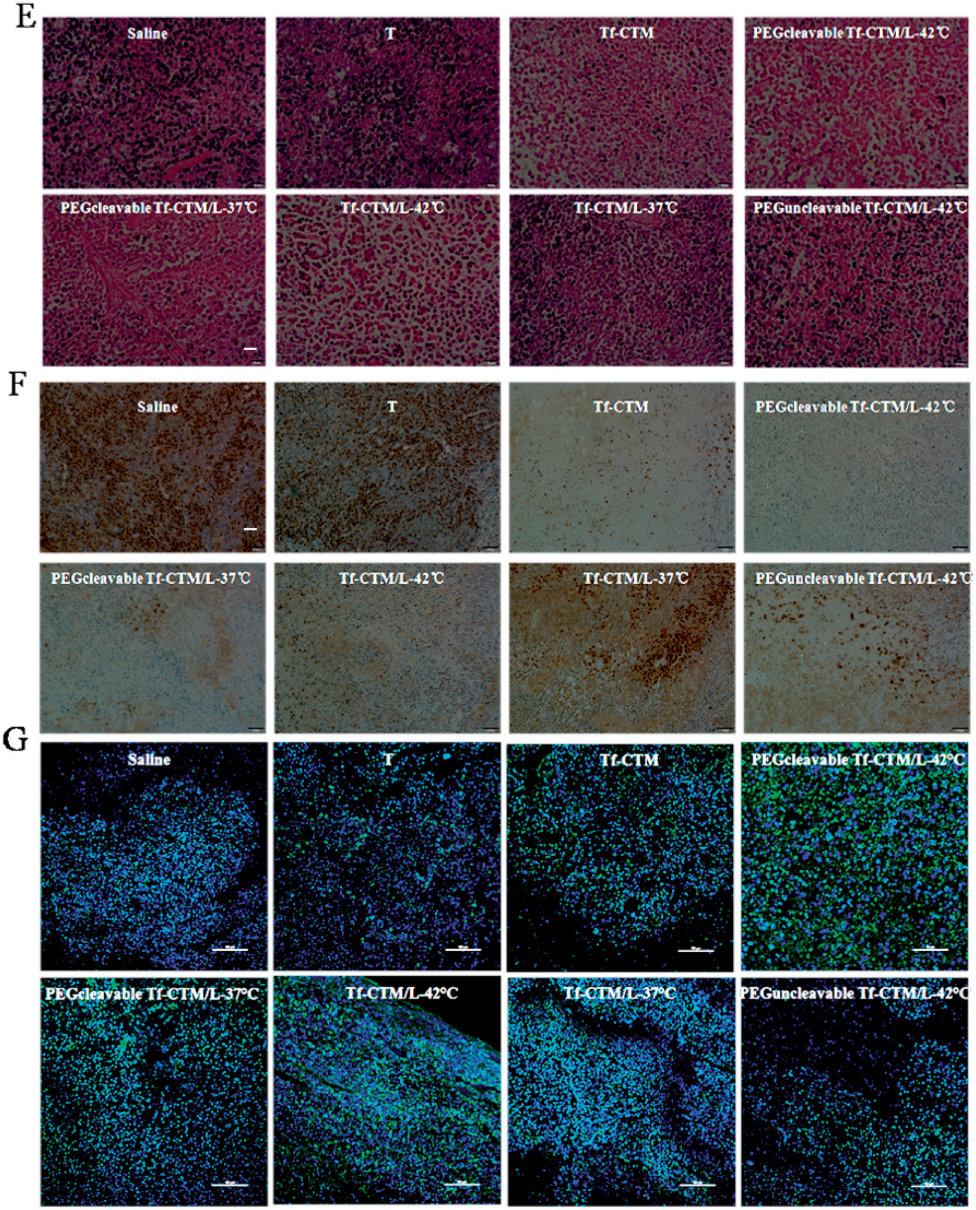

### 3.8. Evaluation of systemic safety

Due to the toxicity, many of the antitumor ingredients were limited to clinical use. The application of tripterine also faces the following obstacles: highly toxic, narrow treatment window, and poor solubility; which severely limits the tripterine to clinical use. PEGcleavable Tf-CTM/L significantly improved the accumulation at tumor sites, while reducing the side effects of non-targeted sites. In this part of the study, we detected the samples of organs and blood to evaluate the potential toxicity of various tripterine treatments. Compared to the normal mice, PEGcleavable Tf-CTM/L-42 °C had a negligible effect on white blood cell (WBC) and platelet (PLT), but slightly reduced the levels of hemoglobin (HGB) and red blood cell (RBC) after 14 days of treatment (Figure 8(A–D)). The liver and spleen index of mice did not change significantly after treatment with PEGcleavable Tf-CTM/L-42 °C (Figure 8(E,F)). In terms of HE staining of main normal organs, PEGcleavable Tf-CTM/L-42 °C hardly caused pathological changes of heart, liver, spleen, lung, and kidney (Figure 8(G)). We also investigated the changes of serum aminopherase including alanineamino transferase (ALT) and aspartate aminotransferase (AST). We also detected the changes of classic indices, including uric acid (UA), blood urine nitrogen (BUN), and creatinine (CREA) of kidney function. As previously reported, tripterine may induce acute damage to the liver and kidney injury.^42-45^ Compared with the normal groups, there was no significant change in the concentrations of ALT, UA, BUN, AST, and CREA among various tripterine treatments, suggesting low toxicity against the function of liver and kidney after treatment with PEGcleavable Tf-CTM/L-42 °C (Supplementary Figure S3).

Figure 8.Evaluation on safety *in vivo*. Level of (A) WBC, (B) RBC, (C) HGB, and (D) PLT in blood samples of mice after 24 h of the last administration. Data are represented as mean ± SD, *n* = 5. (E) Liver index and (F) spleen index of mice treated with different formulations. Data are represented as mean ± SD, *n* = 5. (G) H&E staining images of tissues on each formulation group. Scale bar is 100 μm. (1) Saline, (2) T, (3) Tf-CTM, (4) PEGcleavable Tf-CTM/L (42 °C), (5) PEGcleavable Tf-CTM/L (37 °C), (6) Tf-CTM/L (42 °C), (7) Tf-CTM/L (37 °C), (8) PEGuncleavable Tf-CTM/L (42 °C).

**Figure:**
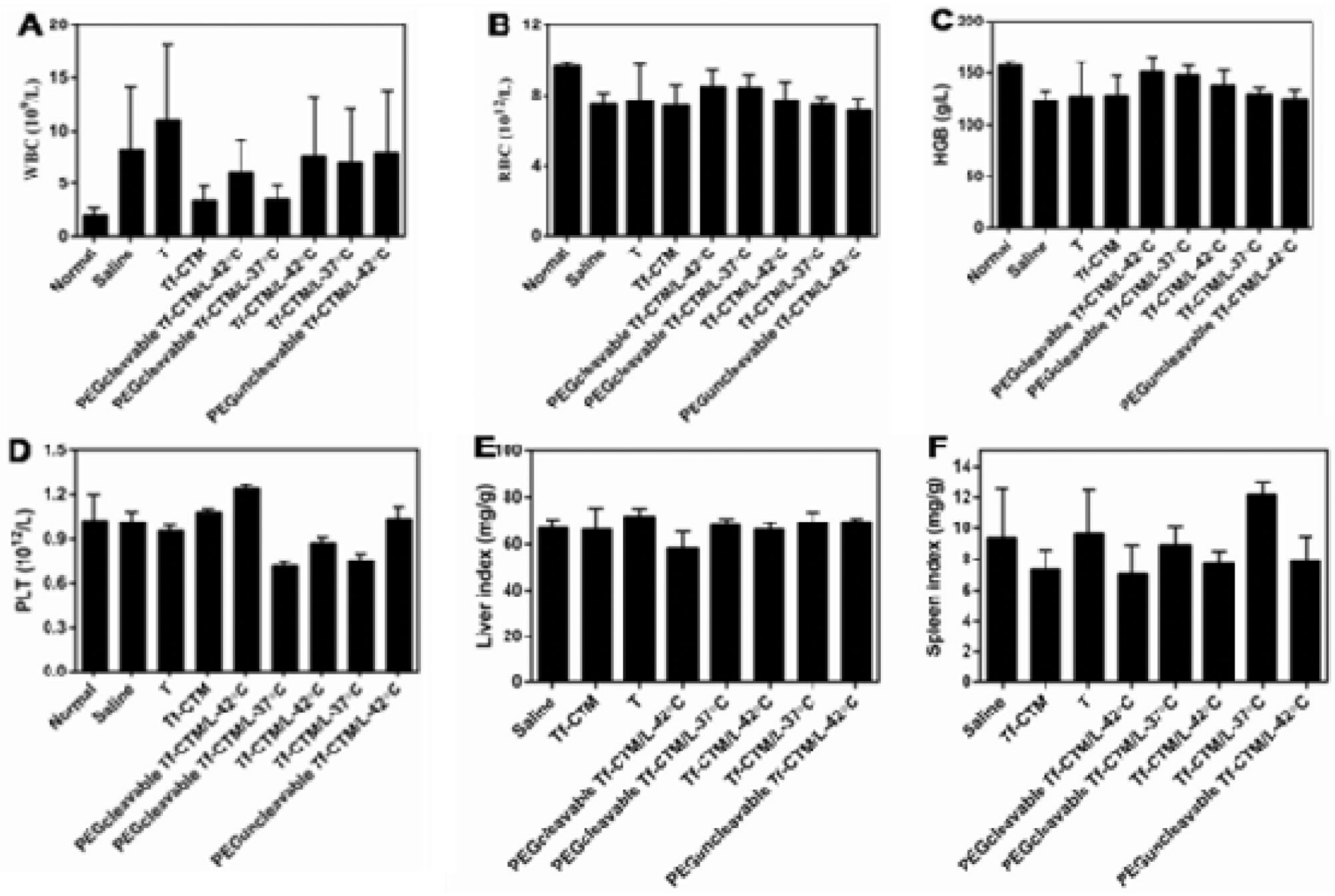

**Figure:**
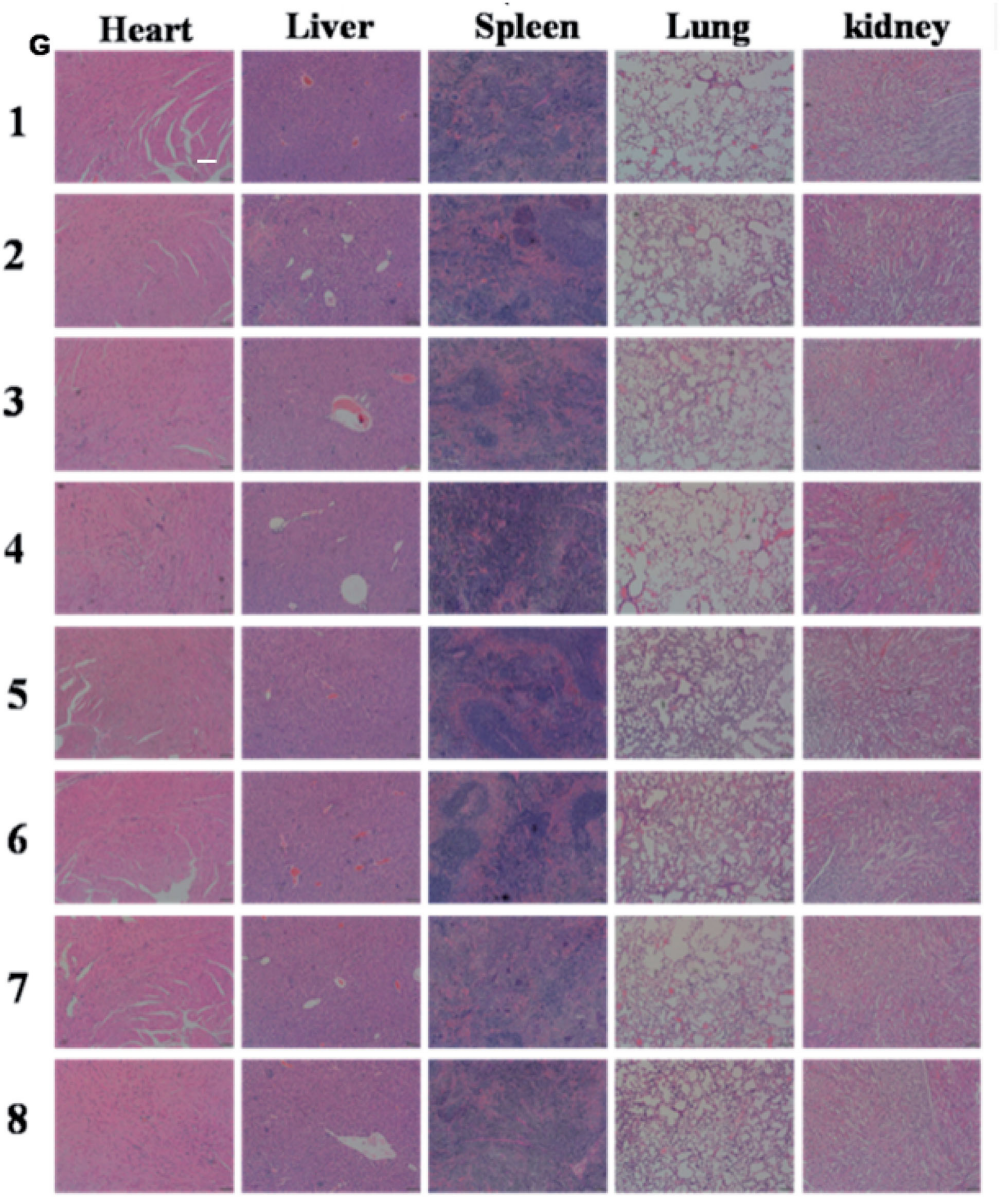

### 3.9. Cytokine determination

The potential mechanism of the anti-tumor effect of PEGcleavable Tf-CTM/L-42 °C was associated with the normalization of the tumor microenvironment by tripterine (Qu et al., 2015, 2017; Chen et al., 2018; Qu et al., 2018). In this part of study, after treated with various tripterine formulations, we detected the alterations of fibroblasts, tumor vessels, and cytokines as well. Cytokines are closely associated with the occurrence and development of cancer. The determination of cytokines in the serum of tumor-bearing nude mice can reflect the antitumor mechanism of various tripterine treatments. It is well known that IFN-γ, IL-2, and IL-12A are capable of inhibiting tumor cell proliferation, blocking the angiogenesis pathway, regulating immunity, and decreasing the formation of tumor-associated fibroblasts (TAFs) (Mumm et al., 2011; Bunimovich-Mendrazitsky et al., 2016; Komohara et al., 2016; Yue et al., 2016; Jiang et al., 2017; Vourc’h et al., 2017; Wu et al., 2017; Ayuthaya et al., 2018; Burkart et al., 2018; Hydes et al., 2018; Kamensek et al., 2018; Lampreht Tratar et al., 2018; Posadassánchez & Vargasalarcón, 2018). In contrast, IL-10, IL-6, CCl2, TNF-α, and TGF-β are considered to be tumor-promoting cytokines, which could promote the proliferation of tumor cells, rebuilding of vascular networks, and so on (Alhamarneh et al., 2015; Song et al., 2015; Lau et al., 2016; Suchal et al., 2016; Kim et al., 2017; Kong et al., 2017). Compared to the saline group, the concentrations of IL-10, IL-6, CCl2, and TGF-β in serum were significantly reduced after treatment with PEGcleavable Tf-CTM/L-42 °C (Supplementary Figure S4A,C,F,H). Interestingly, PEGcleavable Tf-CTM/L-42 °C significantly improved the concentration of IFN-γ compared with the saline group (Supplementary Figure S4B,D,E). There was no noticeable change in the serum level of TNF-α after various tripterine formulations (Supplementary Figure S4G). These results further explain the antitumor mechanism of PEGcleavable Tf-CTM/L-42 °C.

### 3.10. CD 31 assay and α-SMA

**Figure 9.**
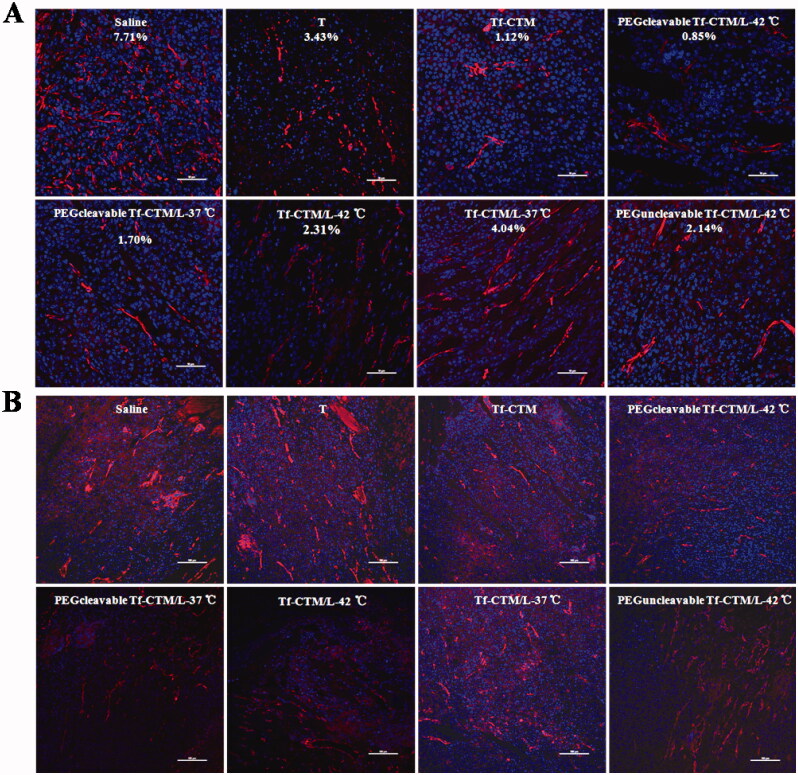
CD 31 and α-SMA Assay (A) Fluorescence images of tumor section stained with anti-α-SMA primary antibody after 24 h of the last administration. The red represents the TAFs and the blue represents nucleus. The bar is 50 μm. (B) Fluorescence images of tumor section stained with anti-CD 31 primary antibody after 24 h of the last administration. The red represents the tumor vessel and the blue represents nucleus. The bar is 50 μm.

TAFs, the major obstacles of deep tumor drug delivery, and fibroblasts were stained using α-SMA. Notably, compared to the saline group, and other tripterine treatments, the level of α-SMA was significantly reduced after treatment with PEGcleavable Tf-CTM/L-42 °C (Figure 9(A)). PEGcleavable Tf-CTM/L-42 °C played a role in suppressing tumor angiogenesis by the efficient accumulation and deep penetration within tumor tissue.

The vascular endothelial cells were conjugated with anti-CD 31 antibody, and then labeled with a secondary antibody (red fluorescence).^45^ After treatment with PEGcleavable Tf-CTM/L-42 °C, the red fluorescence sharply decreased, indicating a significant decrease in vessel density, in addition to indicating the high efficiency of anti-tumor drug accumulation and deep penetration at the tumor sites (Figure 9(B)).

## 4. Conclusions

In summary, we have developed a dual-encapsulation liposomal nanodrug delivery system with two optimal sizes to enhance the efficacy of cervical cancer treatment by enhancing accumulation and penetration at tumor sites. PEGcleavable Tf-CTM/L-42 °C showed stronger inhibition of xenograft tumor growth in comparison with other tripterine treatments. Meanwhile, the near-infrared images of PEGcleavable Tf-CTM/L exhibited a prominent tumor-targeting ability due to prolonged circulation and the EPR effect. Notably, Tf-CTM released from PEGcleavable Tf-CTM/L with small particle size enhanced tumor penetration in HeLa 3D tumor spheroids. More effective accumulation and deep penetration in tumor tissues enhanced the anticervical cancer effects of tripterine. This study offers a new sight and technology to effectively improve the antitumor efficacy of nano-sized anticancer Traditional Chinese Medicine by resolving a contradiction in the optimal size of accumulation and penetration in the tumor sites.

## Supplementary Material

Supplemental Material
