# Supplementary material for: Furin-responsive triterpenine-based liposomal complex enhances anticervical cancer therapy through size modulation
Source: Drug Deliv. 2020 Nov 12;27(1):1608–24. doi: 10.1080/10717544.2020.1827086 (PMC7676817; doi:10.1080/10717544.2020.1827086)
Supplement: Supplemental Material [file IDRD_A_1827086_SM7216.doc]

**Supplementary Information**

**Furin-responsive triterpenine-based liposomal complex enhances anti-cervical cancer therapy through size modulation**

Yunyan Chen^1,2,3^, Mengfei Guo^1,2^, Ding Qu^1,2^,Yuping Liu^1,2^, Jian Guo^1,2^, Yan Chen^1,2^﹡

^1^Affiliated Hospital of Integrated Traditional Chinese and Western Medicine, Nanjing University of Chinese Medicine, Nanjing 210028, China

^2^Jiangsu Provincial Academy of Traditional Chinese Medicine, Nanjing 210028, China

^3^ Wannan Medical College, Wuhu 241002, China

S1. **Synthesis and structure characterization of a kind of furin protease-sensitive material**

**Synthesis and characterization of a kind of furin-sensitive material**

**Synthesis and characterization of mPEG with carboxyl end group (mPEG-COOH)**

500 mg of mPEG (Mn = 5000, 0.1 mmol) and 100 mg of succinic anhydride were dissolved in 5 mL of anhydrous chloroform. Under the protection of nitrogen, the reaction was carried out in presence of 12.2 mg of DMAP (0.1 mmol) as a catalyst for 12 h at 62℃. Next, the react solution was in a rotary evaporator to remove chloroform. Then added 20 mL deionized (DI) water to redissolve and transferred to a dialysis tube (3000 MWCO). After 24 h of dialysis with flowing water, the dialysate was freeze-dried and weighed. Fourier-transformed infrared (FT-IR) spectrum and ^1^H-NMR were used to characterize the structure of mPEG-COOH.

Yield: 75.01%.

mPEG-COOH: ^1^H-NMR (300 MHz, CDCl3): δ3.23 (s, 3 H), 2.64 (s, 2 H). FT-IR: 3468.9 (-OH stretching vibrations), 2887.6 (-CH_2_ stretching vibrations), 1735.8 (-C=O stretching vibrations).

**Synthesis and characterization of maleic anhydride octadecanol**

649.2 mg of octadecanol (2.4 mmol) and 235.2 mg of maleic anhydride (2.4 mmol) were dissolved in 10 mL of toluene. Under the protection of nitrogen, the reaction was carried out for 36 h at 82℃. The crude products were obtained after removing toluene in a rotary evaporator. Then they were purified by silica gel column chromatography with dichloromethane and methanol as eluents (dichloromethane/methanol = 15/1, V /V). High resolution mass spectrometry (HRMS), FT-IR and ^1^H-NMR were used to characterize of the structure of purified products.

Yield: 75.43%

Maleic anhydride octadecanol: FT-IR: 3210.1 (-OH stretching vibrations), 2955.6 (-CH_3_ stretching vibrations), 2918.9 (-CH_2_ stretching vibrations), 1722.6 (-C=O stretching vibrations), 1649.2 (C=C stretching vibrations). HRMS (ESI+) calcd for C_22_H_40_O_4_Na [M+Na]^+^ 391.29266, found 391.28319.


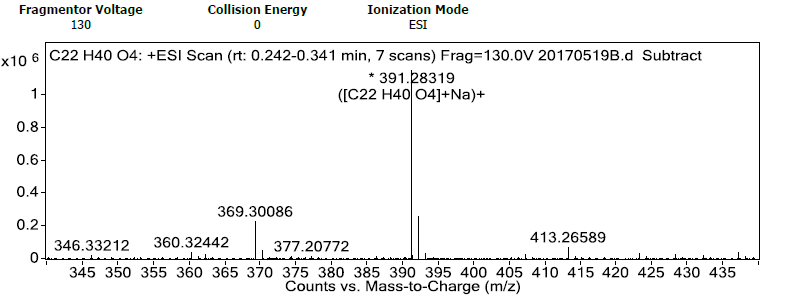
 **Synthesis and characterization of binding products of the peptides with maleic anhydride octadecanol (peptides-maleic anhydride octadecanol)**

40 mg of the peptides (GRVRRSC, 0.048 mmol) and 17.6 mg of maleic anhydride octadecanol (0.048 mmol) were dissolved in 8 mL of anhydrous methanol. Then 10 μL of TEA was added into the reaction. Under the protection of nitrogen, the reaction was carried out for 48 h at room temperature. Finally, the react solution was in a rotary evaporator to remove methanol. Mass spectrometry (MS) and FT-IR were used to characterize of the structure of binding products.

Yield: 77.87%

Peptides**-**-maleic anhydride octadecanol: FT-IR: 3397.5 (-OH stretching vibrations), 2955.7 (-CH_3_ stretching vibrations), 2917.5 (-CH_2_ stretching vibrations), 1679.8 (-C=O stretching vibrations). MS (ESI+): [M+H]^+^ calcd for 1201.7, found 1201.9.

**Synthesis and characterization of mPEG-peptides-maleic anhydride octadecanol**

14.4 mg of peptides-maleic anhydride octadecanol (0.012 mmol) and 61.4 mg mPEG-COOH (0.012 mmol) were dissolved in 5 mL of anhydrous pyridine. Under the protection of nitrogen, the reaction was carried out in presence of 0.2 mg DMAP (0.0012 mmol) and 2.7 mg DCC (0.0132 mmol) for 72 h at room temperature. Then added 40 mL deionized (DI) water to the reaction solution and transferred to a dialysis tube (3000 MWCO). After 24 h of dialysis with flowing water, the dialysate was by suction filtration and freeze-dried. The final product was characterized by FT-IR.

Yield: 82.59%

mPEG-peptides-maleic anhydride octadecanol: FT-IR: 3419.1 (-OH stretching vibrations), 3276.1 (-NH stretching vibrations), 2888.5 (-CH_2_ stretching vibrations), 1732.0 (-C=O stretching vibrations), 1621.9 (amide I band), 1533.6 (amide Ⅱ band).


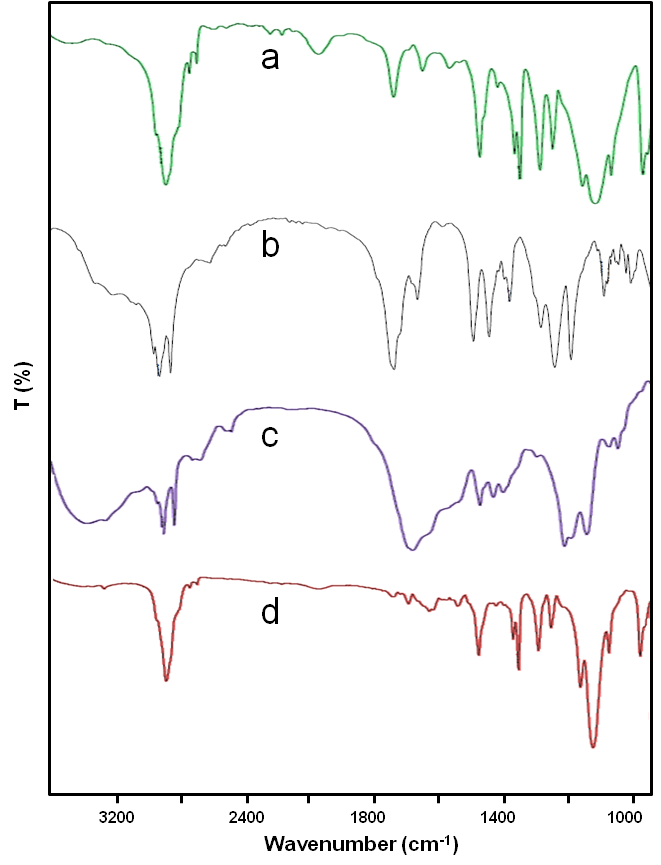


FT-IR spectral analysis data, where (a) mPEG-COOH, (b) maleic anhydride octadecanol,(c) peptides-maleic anhydride octadecanol and (d) mPEG-peptides-maleic anhydride octadecanol.


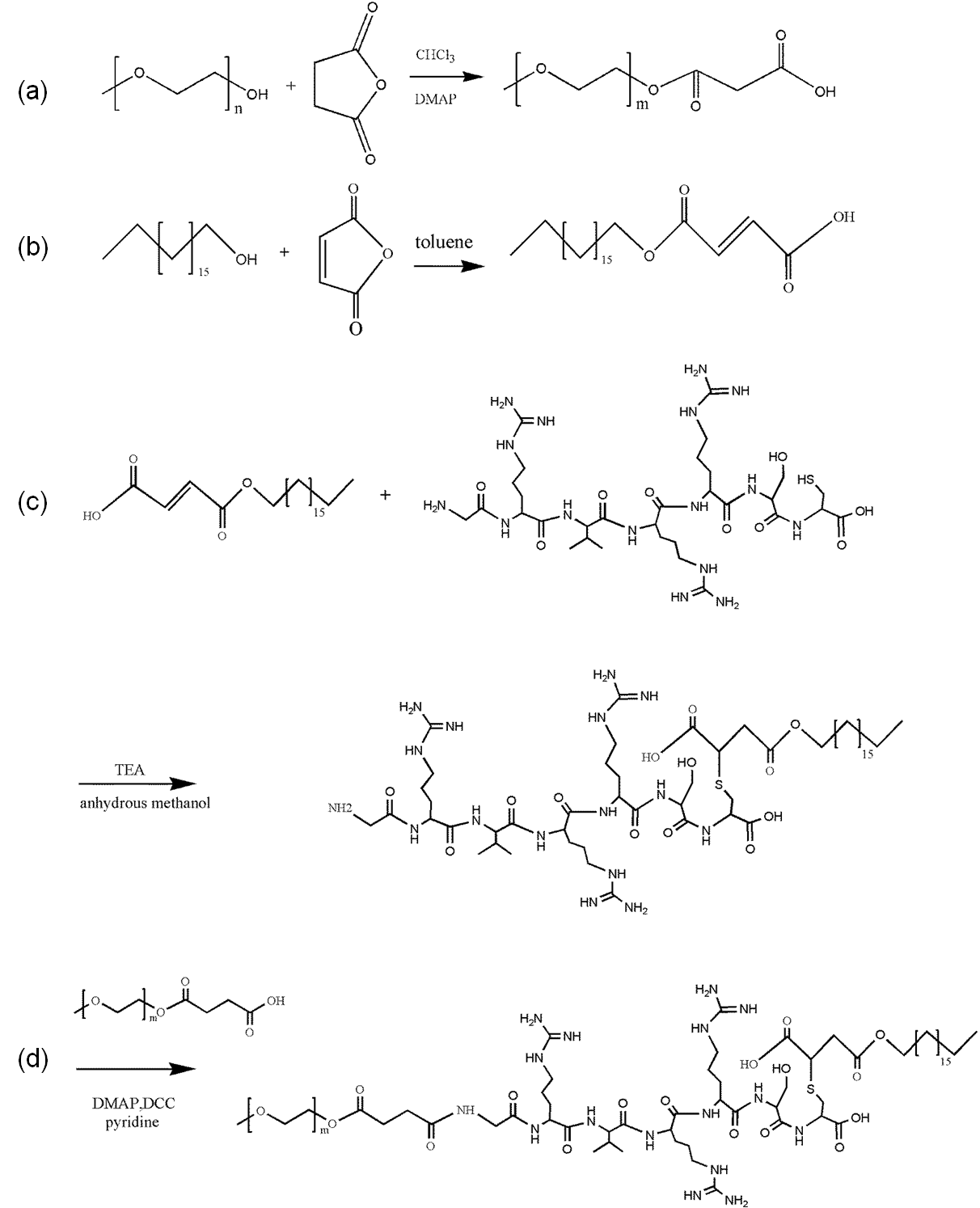


Synthetic route of (a) mPEG-COOH, (b) maleic anhydride octadecanol,(c) peptides-maleic anhydride octadecanol and (d) mPEG-peptides-maleic anhydride octadecanol.

S2.
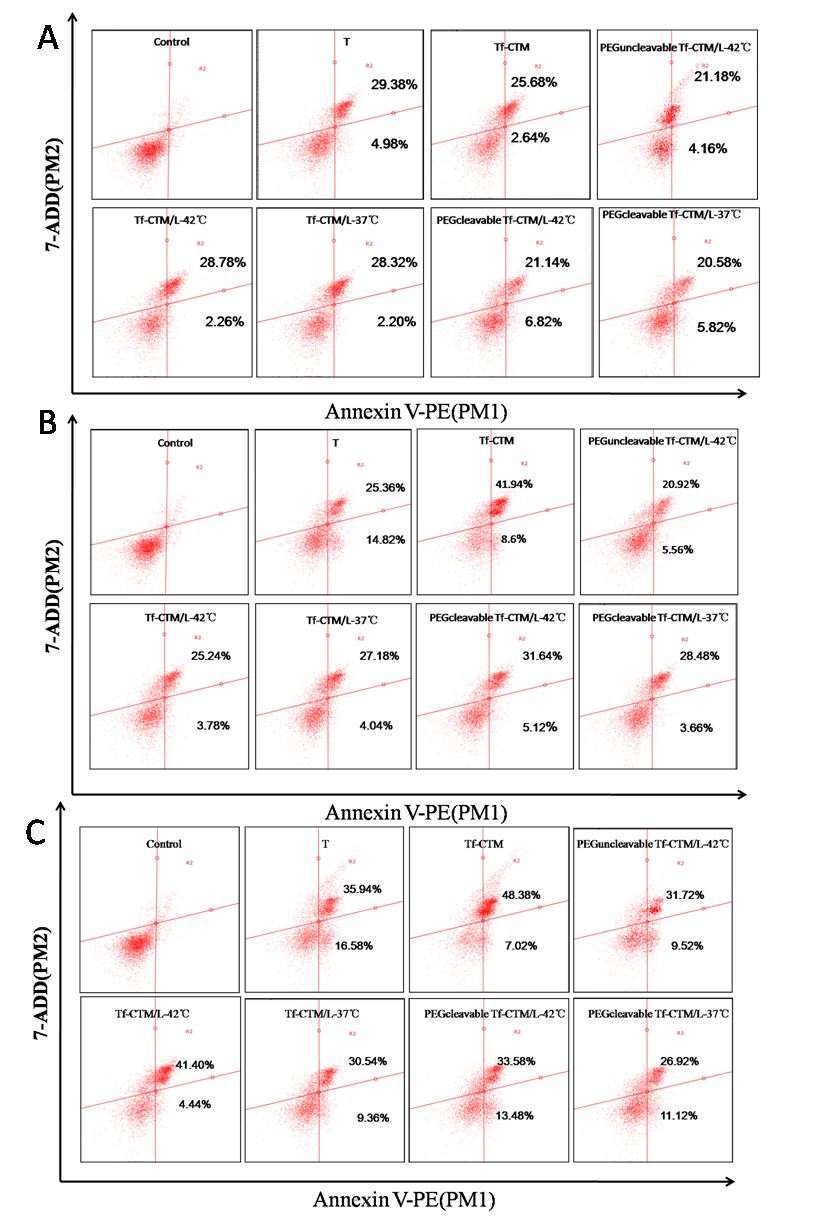


Figure S2. Apoptosis rate of HeLa cells. The drug concentration was 1 μg/mL and the administration time was 2 h (A), 4 h (B), 6 h (C).

S3.


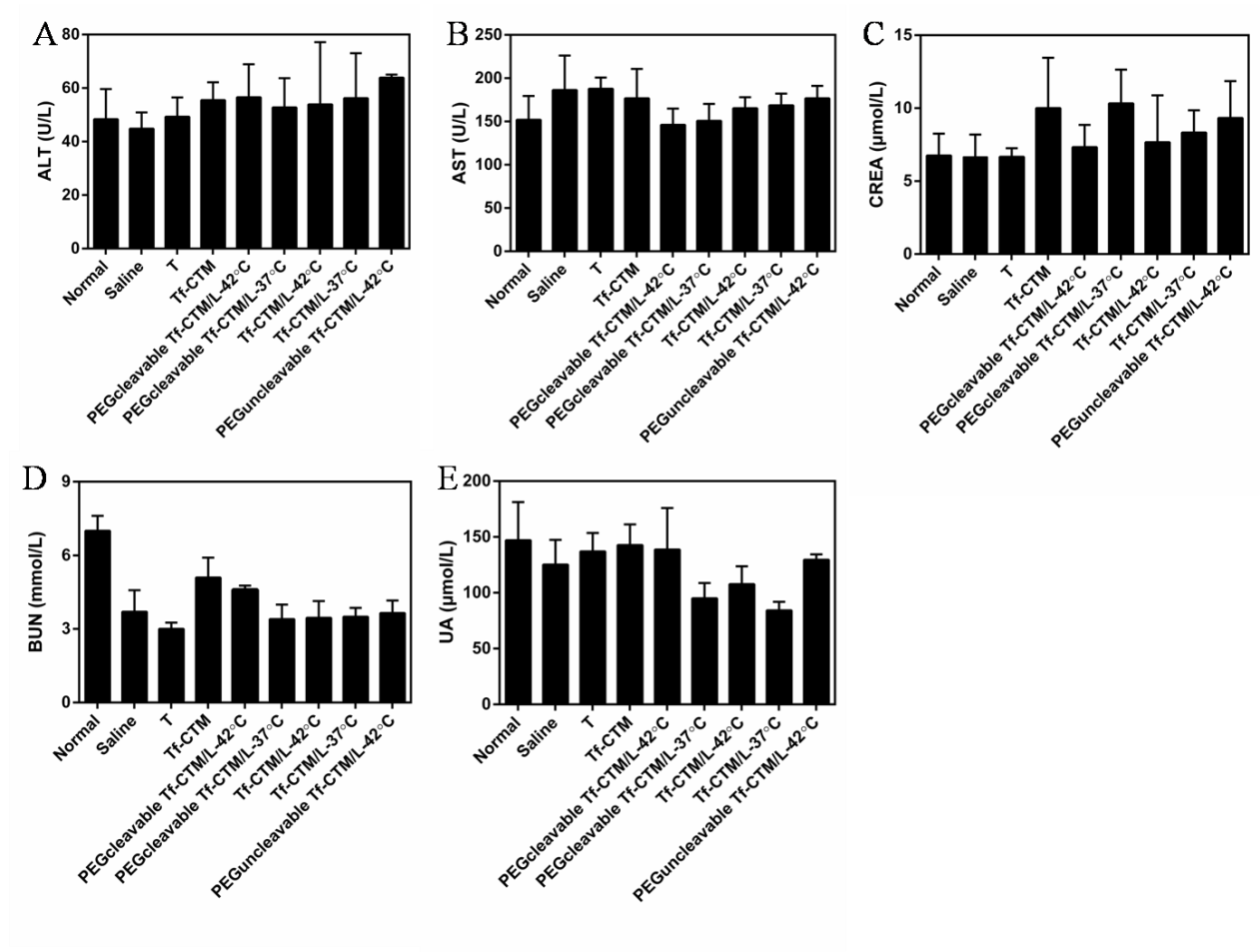


Figure S3. Liver and kidney function indicies among nude mice（n = 5, ± *s*）

(A)ALT (B) AST (C) CREA (D) BUN (E)UA


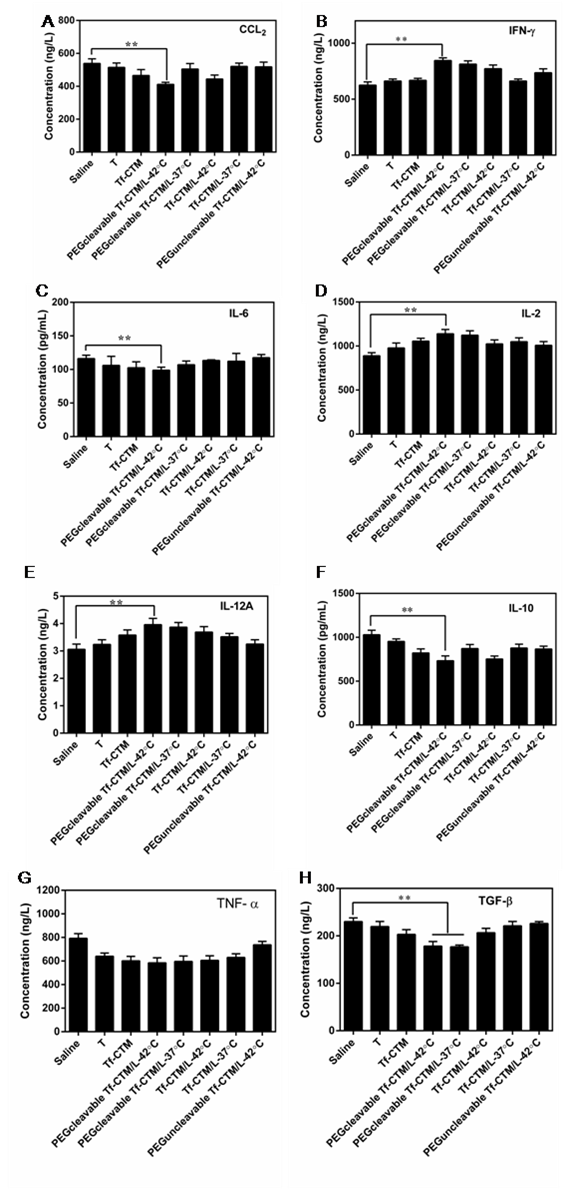
S4.

Figure S4. Serum level of (A) CCL2 (B) IFN-γ (C) IL-6 (D) IL-2 (E) IL-12A (F) IL-10 (G) TNF-α (H) TGF-β of mice after 24 h of the last administration. Data are represented as mean ± SD, n = 6. ^**^*P* <0.01.

S5.


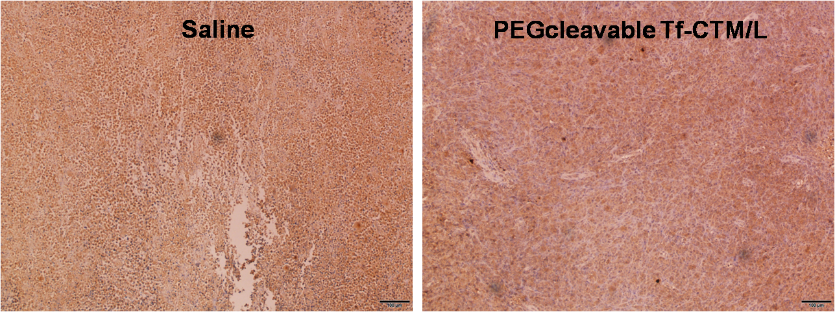


Figure S5. Immunohistochemical images of tumor sections stained with anti-Furin.

The bar is 100 μm.
